# Simple Imaging System for Label-Free Identification of Bacterial Pathogens in Resource-Limited Settings

**DOI:** 10.1155/2024/6465280

**Published:** 2024-11-19

**Authors:** Clément Douarre, Dylan David, Marco Fangazio, Emmanuel Picard, Emmanuel Hadji, Olivier Vandenberg, Barbara Barbé, Liselotte Hardy, Pierre R. Marcoux

**Affiliations:** ^1^Laboratoire d'Électronique et de Technologie de l'Information, French Alternative Energies and Atomic Energy Commission, Grenoble, France; ^2^Institut de Recherche Interdisciplinaire de Grenoble, French Alternative Energies and Atomic Energy Commission, Grenoble, France; ^3^Research and Technology Innovation Unit, Laboratoire Hospitalier Universitaire de Bruxelles-Universitair Laboratorium Brussel (LHUB-ULB), Université Libre de Bruxelles (ULB), Brussels, Belgium; ^4^Center for Environmental Health and Occupational Health, School of Public Health, Université Libre de Bruxelles (ULB), Brussels, Belgium; ^5^Department of Clinical Sciences, Institute of Tropical Medicine, Antwerp, Belgium

**Keywords:** bacterial identification, deep learning, lensless imaging

## Abstract

Fast, accurate, and affordable bacterial identification methods are paramount for the timely treatment of infections, especially in resource-limited settings (RLS). However, today, only 1.3% of the sub-Saharan African diagnostic laboratories are performing clinical bacteriology. To improve this, diagnostic tools for RLS should prioritize simplicity, affordability, and ease of maintenance, as opposed to the costly equipment utilized for bacterial identification in high-income countries, such as matrix-assisted laser desorption/ionization time-of-flight mass spectrometry (MALDI-TOF MS). In this work, we present a new high-throughput approach based on a simple wide-field (864 mm^2^) lensless imaging system allowing for the acquisition of a large portion of a Petri dish coupled with a supervised deep learning algorithm for identification at the bacterial colony scale. This wide-field imaging system is particularly well suited to RLS since it includes neither moving mechanical parts nor optics. We validated this approach through the acquisition and the subsequent analysis of a dataset comprising 252 clinical isolates from five species, encompassing some of the most prevalent pathogens. The resulting optical morphotypes exhibited intra- and interspecies variability, a scenario considerably more akin to real-world clinical practice than the one achievable by solely concentrating on reference strains. Despite this variability, high identification performance was achieved with a correct species identification rate of 91.7%. These results open up some new prospects for identification in RLS. We released both the acquired dataset and the trained identification algorithm in publicly available repositories.

## 1. Introduction

Appropriate treatment of patients suffering from bacterial infections requires a *diagnosis* step, i.e., the identification of the bacterial species at hand [1]. Diagnostic laboratories operating in resource-limited settings (RLS) face many challenges [2, 3] such as logistics, equipment, and infrastructure: Only 1.3% of the sub-Saharan African diagnostic laboratories have the capability to perform such clinical bacteriology [4]. To improve this, diagnostic tools in RLS should be simple, affordable, and maintenance-friendly. Unlike in industrialized countries, microbial identification such as the matrix-assisted laser desorption/ionization time-of-flight mass spectrometry (MALDI-TOF MS) technology is poorly suited for RLS, as the need for complex instrumentation and expensive maintenance makes it impractical for implementation outside of large and well-equipped laboratories. Biochemical testing can be compatible with RLS requirements [5], but the time-to-result is long, as not only it is a grown culture necessary to initiate the test procedure but also that procedure can then subsequently extend beyond 20 h. Moreover, biochemical testing requires the usage of specific consumables.

Optical identification methods, on the contrary, have many advantages for RLS or isolated settings: They are label-free [6] and applicable to single cells [7, 8] and microcolonies [9], as well as to colonies [10, 11]. Among those methods, lensless imaging [12] is emerging as it allows for the fast analysis of a large area of the Petri dish. Lensless imaging consists in placing the sample to be analyzed as close to the image sensor as possible (< 1 mm), without the use of any intervening optical objectives. This imaging technique can only be employed for thin and nondiffusive samples, thereby excluding blood-supplemented media. However, it presents several advantages: Firstly, no optical aberrations, such as image distortion, can be caused by the use of lenses. Furthermore, since the pixel pitch of modern image sensors is as low as a few micrometers, lensless imaging can resolve structures of a few dozen micrometers. Finally, as the sample is very close to the image sensor, the magnification is equal to one, which means that a wide field of view (FoV), i.e., at the mm^2^ scale, is available for imaging the sample, much larger than the ones used in conventional optical microscopy (*μ*m^2^ scale). This technique has been used to monitor eukaryotic cell cultures [13, 14], to identify and enumerate bacterial colonies [15, 16], and to observe and analyze lysis plaques of eukaryotic [17] or prokaryotic [18] viruses.

**Figure 1:**
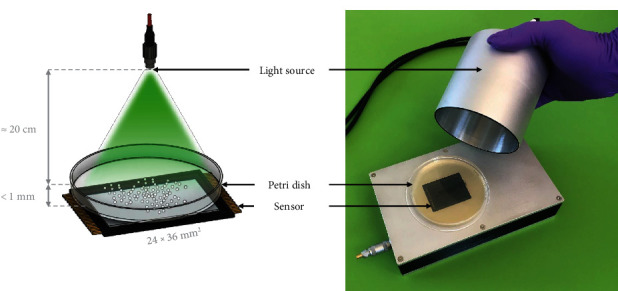
Picture of our proposed wide-field lensless imaging system, with an uninoculated Petri dish placed on the sensor.

In this work, we introduce a lensless imaging system (Figure 1) whose key asset is its very large FoV 24 × 36 mm (864 mm^2^), more than 20 times larger than the other wide-field lensless imaging systems proposed for bacterial identification [19, 20, 21], while still maintaining a low pixel size, comparable to those systems (3.76 *μ*m) for high-resolution imaging. Such a wide field allows for the imaging of a significant portion of the Petri dish in one acquisition, without any biological labelling, enabling a truly high-throughput colony identification process. Although some lensless imaging systems currently in existence include mechanical components allowing the scanning of the entire Petri dish [20, 21], these systems are slower and more expensive, making them less suitable for deployment in RLS.

While lensless imaging stands out as a promising technique for bacterial identification in RLS, identification performance based on images acquired by such systems still requires evaluation. Decreasing the coherence of the illumination compared to laser-based methods [22] inherently degrades the quality of image features. Moreover, crucially, no bacterial identification study using lensless imaging has been reported on clinical isolates sampled from patients. Previous studies have been limited to reference strains [16, 19, 20, 21], often with a low number of strains (sometimes just one per species), potentially leading to overly an optimistic assessment of identification performance. Indeed, in a clinical setting with a large sample size, each species comprises as many strains as there are patients, introducing significant variability within the same species. Therefore, clinical studies enrolling a large number of patients can offer a much more realistic assessment of the identification performance of lensless imaging.

**Figure 2:**
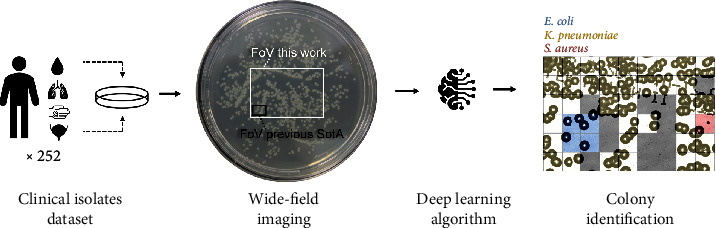
General pipeline of our study. The previous state-of-the-art mentioned in this figure is elaborated in [19, 20].

Here, we report the very first clinical study of lensless imaging for label-free optical identification of bacterial colonies growing on agar, using a wide-field imaging device (Figure 2). We collected a database of 252 clinical bacterial isolates, focusing on five species chosen among the bacterial isolates most frequently encountered in clinical bacteriology laboratories operating in RLS. The imaging system's wide FoV enabled us to efficiently populate each category with thousands of examples.

For a bacterial species identification process to be truly high-throughput, the data acquisition step and the data analysis step needed to be streamlined in order to provide a timely diagnosis with minimal human intervention. Hence, a natural counterpart to a high-capacity imaging system was an automated classification algorithm. Consistent with the latest advancements in the computer vision field, we developed and trained a deep learning model for species identification. As we assessed its performance, we meticulously investigated the reasons behind any misclassifications in order to draw perspectives regarding how to improve automatic identification of bacterial colonies in these challenging clinical settings.

In summary, our contribution encompasses three key aspects, each addressing different stages of the bacterial identification process:

• We propose a new lensless imaging system with a large FoV devoid of mechanical components,
• We employ this system to acquire a large dataset of bacterial colonies from clinical isolates, and
• We validate this process through the development and training of a deep learning model for the identification of bacterial species.

## 2. Results

### 2.1. Acquired Images

Table 1 presents the breakdown of species and specimen type in the dataset. Figure S1 specifies the phylogenetic relationships between each of the five species.

**Figure 3:**
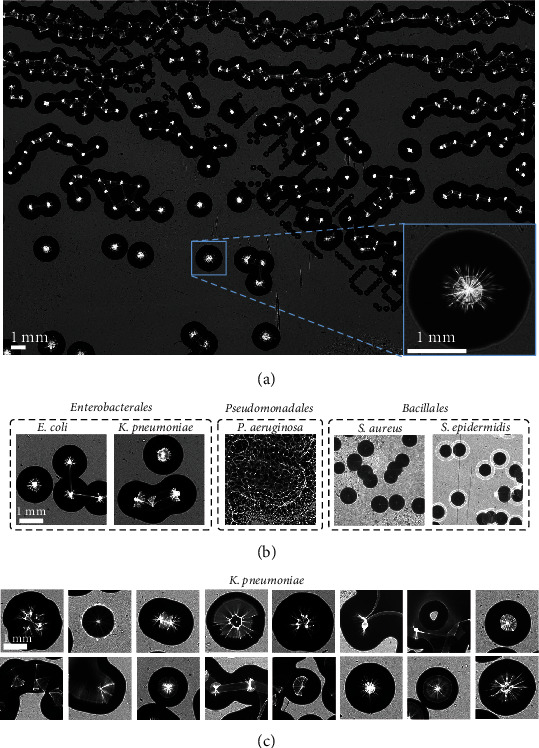
Some examples of images from the dataset acquired with the lensless imaging system. (a) An acquisition of *E. coli* colonies originating from a blood sample. Colonies appear as dark disks with a bright pattern. The smaller dark spots populating the downward diagonal of the image correspond to engraved markings at the back of the Petri dish. Bottom left of panel (a): a zoomed-in visualization of a specific colony from the full image. The bright web-like pattern is the scatterogram. (b) Most common morphotypes (in our dataset) of each of the studied species. All images are at the same scale, indicated on the left image. We increased the contrast of the *Bacillales* images to better display the scatterograms, which appear fainter than for the two other families. (c) Colonies from various acquisitions of *K. pneumoniae* blood samples. Some of the colonies shown come from the same acquisition. All images are at the same scale, indicated on the top-left image.

Several examples of acquired images are presented in Figure 3. At the image scale (Figure 3(a)), one can immediately observe the significant number of colonies that can be imaged in a single acquisition. Figure S2 shows other examples of full images acquired by the system, in higher resolution. One can also note the strong variability of colony density within a given image, with areas where colonies are heavily clustered to the point where individual colonies cannot be distinguished (top of Figure 3(a)). This density variability is due to our plating protocol, reflecting true field conditions where the bacteria species and concentration are unknown before plating, and the resulting colony density is necessarily less controlled (see Section 5.2).

At the colony scale, the bottom-left part of Figure 3(a) presents an example of a single *E. coli* colony. The most glaring features of a colony are its general shape and size, but lensless imaging also allows for the acquisition of more subtle features. Indeed, because colonies form a dome-like shape and are composed of bacteria transparent to visible light, they share some optical properties with lenses, changing the path of incoming light rays [22]. Hence, a coherent light ray leads to the apparition of a bright diffraction pattern (*scatterogram*) inside the colony, whose shape is dependent on the morphological characteristics of the individual bacteria and on the spatial organization of the colony [19].

To assess the feasibility of identifying the species of the acquired colonies, the most important consideration was the extent of the cross-species and within-species variability of colony morphotypes (shape, size, and scatterogram). Regarding cross-species variability, Figure 3(b) presents the most common species morphotypes in our dataset. These morphotypes were (i) significantly different from one order to the next (i.e., *Enterobacterales* colonies appear very different from *Bacillales* ones, which themselves appear different from *Pseudomonadales* ones) but (ii) quite close within a given order (i.e., within the *Enterobacterales* order, *E. coli* colonies resembled *K. pneumoniae* ones, and within the *Bacillales* order, *S. aureus* colonies resembled *S. epidermidis* ones). Hence, to discriminate species within a given order, we relied on the capability of the classification algorithm to exploit more subtle features than the ones immediately apparent, such as the structure of scatterograms.

However, species identification, whether across or within orders, was significantly complicated by the high variability of colony morphotypes *within a given species*. As each clinical isolate represented a different bacterial strain, variations in morphotypes and growth rate were to be expected across acquisitions. Some isolates were so visually significantly different from the species' “typical” morphotype that we conjectured them to be morphotypic variants, as such variants appear in other works focusing on clinical isolates [23, 24]. Moreover, we even observed strong *within-strain* variability. Indeed, varying colony density in a given acquisition led to strong variations in colony size due to the competition for nutrients by neighboring colonies [19] (as can be seen by comparing the top and bottom halves of Figure 3(a)). It is noteworthy that scatterograms were particularly influenced by this variability in growth, as their aspect significantly depends on the colony size. In addition, in the case of high density and clustered colonies, light going through two adjacent colonies behaved differently than when going through a single one (as can be seen by the light “bridges” between colonies on clustered colonies in Figure 3(a)).

Because of all of these sources of variability, colonies of the same species and even of the same strain could nevertheless show very different morphotypes (Figure 3(c)). Hence, the data at hand was considerably more multifaceted than the one gathered by controlled reference strains studies, but the challenge that it represented for automatic identification reflected the hurdles that microbiologists face in field conditions.

### 2.2. Identification

Since clinical samples can possibly be polymicrobial, species identification was required at the colony scale. To address this requirement, we separated each image into a regular grid of small square patches, which were then individually processed by a supervised neural network (Figure 2, right). Patching the image regularly like so allowed us to seamlessly process both individual and clustered colonies, a necessary feature for our dataset mimicking field conditions (see Section 5.3.1 for more details).

**Figure 4:**
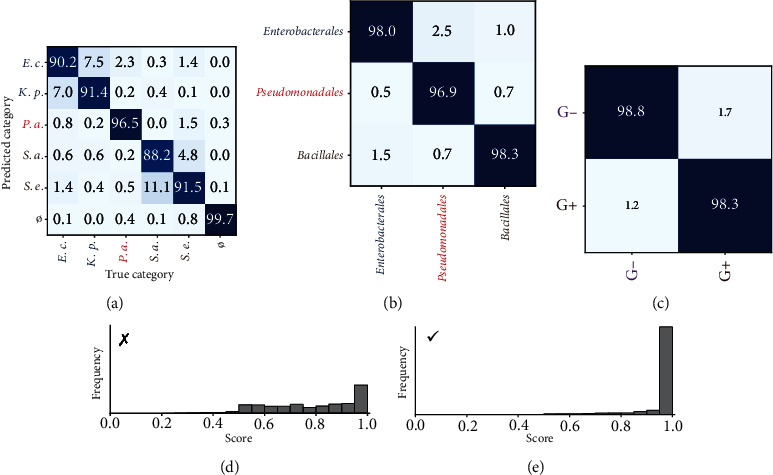
Classification results. (a) Confusion matrix presenting the classification accuracy of the algorithm on the dataset, at the species level. The matrix is expressed in percentages and normalized by columns, i.e., true populations of each category. (b) Confusion matrix at the order level, leaving out the empty patches. (c) Confusion matrix at the gram level, leaving out the empty patches. (d) Histogram of scores for incorrectly classified patches at the species level. (e) Histogram of scores for correctly classified patches at the species level. Both histograms are normalized, i.e., their total areas are equal.

The confusion matrix depicting the network's predictions, obtained through cross-validation, is presented in Figure 4 for several taxonomic levels. Upon initial examination, the classification accuracy of the *No colony* case in Figure 4(a) validates the algorithm's ability to distinguish bacteria from the Petri dish background (98.2% precision and 99.6% recall for this task). Focusing solely on patches with colonies, the classification accuracy at the bacterial species level was 91.7%. Notably, most misclassifications (74.8%) were within-order errors. Hence, classification at the order level was possible with 97.9% accuracy (Figure 4(b)). On a broader scale, classification at the gram level was possible with 98.6% accuracy (Figure 4(c)).

If we consider the identification accuracy at the species level, our correct identification rate of these five species (91.7%) is comparable not only to the average identification rate (92.7%–93.2%) by mass spectrometry—a method that, similarly to our study, requires a bacterial culture to carry out identification—met in our routine clinical laboratory activity on 52 bacterial species [25], but also to the 90.9%, reported in a meta-analysis about MALDI-TOF MS for bacterial identification [26].

Most bacterial identification methods provide the clinician with some measure of certainty associated with the prediction, called a *score*. While studies implementing neural networks for classification tasks do not routinely report such scores, these architectures inherently possess a means to express some form of certainty. Indeed, for a given input, the output of a neural network trained for closed-set classification is a probability distribution over all possible categories in the training set. Therefore, even though these probability distributions may contain artifacts [27], the probability associated with the most probable category (i.e., the category that the network predicts) can act as a reasonable measure of certainty.

Figures 4(d) and 4(e) show the histograms of scores computed this way, in the case of correct (Figure 4(d)) and incorrect (Figure 4(e)) predictions at the species level. We can observe that scores coupled with correct predictions are significantly higher than those relative to wrong predictions. For example, 84% of correctly predicted patches were classified with over 95% confidence, while this was only the case for 27% of incorrectly predicted patches. These results demonstrate that this score is indeed correlated with the accuracy of predictions and legitimize its usage as a metric to trust the output of the algorithm, as it is done with other bacterial identification methods.

### 2.3. Explainability Analyses

To better understand the training process of the network and to draw perspectives on how to mitigate misclassifications, we conducted several post hoc analyses, i.e., analyses carried out after the prediction step.

#### 2.3.1. Misclassifications

**Figure 5:**
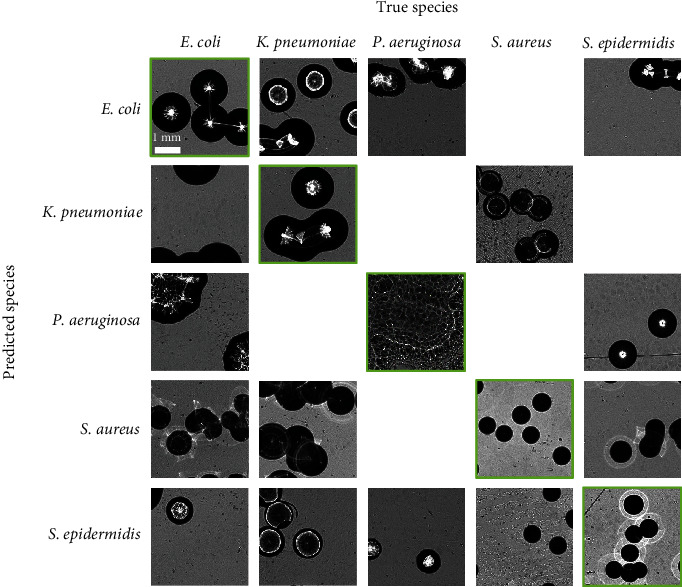
Misclassifications. Some representative examples of the “predicted species–true species” pairs, presented in the form of a confusion matrix. Images with green borders correspond to correct classifications, all others to misclassifications. We limited this visualization to cases where these pairs represented a sufficient proportion of the total dataset (arbitrarily set to 0.1%). All images are at the same scale, indicated on the top-left image.

As a first straightforward analysis, we parsed all of the misclassified patches in an effort to unearth underlying patterns.  Figure 5 gives a representative example for each mismatched “predicted species–true species” pair.

Cross-order misclassifications seemed to be mainly caused by strongly atypical morphotypes. See, for example, the *E. coli* and *P. aeruginosa* columns where colonies could be significantly smaller than the typical morphotype of their species (*S. aureus* and *S. epidermidis* line) or vastly different from it (*P. aeruginosa* line), to the point of being visually closer to their (wrongly) predicted species.

Regarding within-order misclassifications (i.e., *E. coli ↔ K. pneumoniae* and *S. aureus ↔ S. epidermidis*), misclassified patches showed colonies with typical morphotypes. For most of these patches, we were unable to identify specific patterns when comparing them to correctly classified patches. The only clear trend that appeared more frequently than its occurrence in the whole dataset was related to the patching process. This process had led to the creation of some patches containing fragments of colonies with no visible scatterograms and, consequently, little feature information for the algorithm to exploit (see the patch of *E. coli* predicted as *K. pneumoniae* in Figure 5). Further discussion regarding this edge case and strategies to mitigate its detrimental effect is discussed in section S3.2.

#### 2.3.2. Network Focus

**Figure 6:**
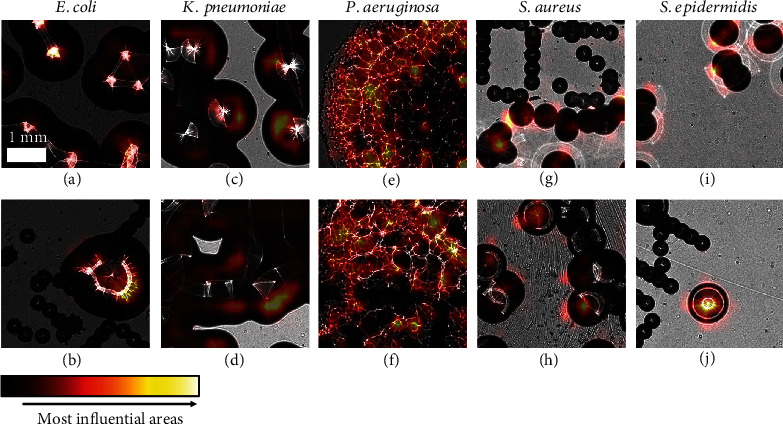
Attribution study. Two selected representative patches for each species (columns) with their corresponding attribution heat maps superposed. Heat maps are colorized with the “hot” colormap (bottom), i.e., highly influential areas for the network's choice are indicated with a yellow hue. (a–j) All images are at the same scale, indicated on the top-left patch.

To gain further insight into our network's *modus operandi*, we conducted an *explainability study* [28] to highlight the most important image features from the network's standpoint. Deep learning methods are often qualified as “black boxes” [29] as the relationship between the input and the output is not easily understandable. As a result, numerous methods have been developed to aid in the interpretation of neural networks. One such category is *attribution methods*, which highlight, for a trained neural network and a given input image, the regions within that image that exerted the most influence on the network's prediction. We implemented a widely spread attribution method specifically designed for convolutional networks [30] (Figure 6).

While this figure only shows a limited number of patches, we found that the features strongly influencing the network's decision were highly consistent within a given species across the dataset. For example, regarding *E. coli*, *P. aeruginosa*, and large enough *Staphylococcus* colonies, the network generally focused on the scatterograms (Figures 6(a), 6(b), 6(e), 6(f), 6(h), and 6(j)), thus confirming our intuition that these structures carried relevant discriminative features for species classification. Regarding smaller *Staphylococcus* colonies with no clear scatterogram, the network seemed to focus on features extracted from the colony borders (Figures 6(g) and 6(i)). Perhaps, the most surprising result was for *K. pneumoniae* where the network frequently focused not on scatterograms but rather on darker areas of the colonies (Figures 6(c) and 6(d)). This result supported the hypothesis of the presence of discriminative features in dimmer areas of colonies, invisible to the human eye. Finally, it is noteworthy that when we acquired the first images for this study, we were concerned about the presence of the markings on the back of Petri dishes (see Figure 3(a)) as they led to structures of similar shape and size and contrast to *Staphylococcus* colonies. Visualizations like the ones of Figures 6(g) and 6(j) were reassuring regarding the network's resilience to those artifacts, as it seemed that the network focused exclusively on the bacterial colonies even when inscriptions were present.

#### 2.3.3. Exploration of Feature Space

**Figure 7:**
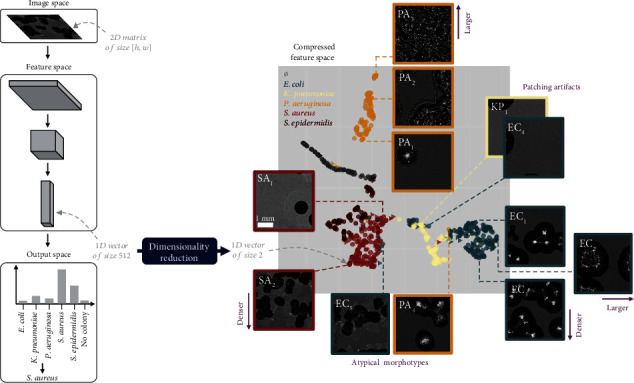
Feature space exploration. Left: Simplified diagram of the classification process of a neural network. In this analysis, we focused on intermediate features before the output space, which we projected to a smaller dimension. Right: Compressed feature space (i.e., after dimensionality reduction) populated with projected features extracted from 640 randomly sampled patches from the test set. No scale is provided, as the absolute values of the compressed space's axis are meaningless. The color of a point corresponds to the label of the corresponding input patch. The shape of a point represents the correct (circle) or incorrect (triangle) final classification of that patch by the network. We show, for some selected points (representative of their neighborhood in feature space), the corresponding input patch. We indicate in purple some image-space tendencies identified by parsing these patches.

Finally, we dove deeper into the network's inner workings by exploring its representation of images in feature space. While the confusion matrix provided valuable insights regarding how the network segmented the data between species, this examination only focused on the final step of the network's classification process. However, a trained neural network essentially acts as a series of consecutive feature extraction operations (Figure 7, left), and the analysis of intermediate features can shed light on which image characteristics have a true impact on the trained network's internal representation of data [31]. We focused on the features computed immediately before the network calculated the category distribution of the input (i.e., located just after the last convolutional layer of the network). These semantically rich features are often studied in network feature analysis [32] as they represent the high-level information that the network then exploits for the classification process. These features are in the form of an extended one-dimensional vector (512 features in our case): We applied an unsupervised nonlinear dimensionality reduction technique [33] to project this data to two dimensions. The resulting visualization allowed for the identification of meaningful patterns in this feature space (Figure 7, right).

From an interspecies standpoint, a first glance at this feature space confirmed the results from the species confusion matrix (Figure 4(a)). In particular, we could observe clear clusters corresponding to the different species, in line with the high accuracy obtained by the network. Additionally, the two cluster pairs corresponding to bacteria from the same order (*S. aureus* and *S. epidermidis*, bottom left, and *E. coli* and *K. pneumoniae*, bottom right) were near one another and somewhat mixed, and conversely, the most isolated cluster corresponded to the species easily distinguished from the rest of the dataset (*P. aeruginosa*, top).

To obtain more actionable insights into which image features held true importance from the network's perspective, it was of great interest (i) to parse, for each point in feature space, the corresponding input patch and (ii) to conduct that analysis within each cluster (i.e., an intraspecies analysis). This process allowed the discovery of some trends: We found that the axes of given clusters (in feature space) were aligned with the evolution of some traits of the patches (in image space) (purple annotations in Figure 7), indicating that these image features heavily determined the network's internal representation of data and ultimately the classification accuracy. In particular, we observed that the principal axes of the *E. coli*, *P. aeruginosa*, and *S. aureus* clusters followed the variation of the density and size of the colonies (see the relative positions of EC1 → EC2, EC1 → EC3, PA1 → PA2 → PA3, and SA1 → SA2). Another interesting insight was the presence of a cluster of patches suffering from patching artifacts (KP1 and EC4, right), showing that these patches were indeed considered separately from the rest of the dataset. Finally, unsurprisingly, patches containing colonies with morphotypes so atypical that they strongly resembled another species produced a feature vector that was contained in the cluster of that species (EC5 in the *S. aureus* cluster, PA4 in the *E. coli* cluster, bottom).

## 3. Discussion

### 3.1. Comparison to Other Optical Bacterial Identification Methods

The task of bacterial identification can be undertaken at several stages in the development cycle of bacteria. A number of optical bacterial identification methods are able to operate at the single-cell scale without a need for a bacterial culture [34, 35]. These methods have a very low time-to-response (on the minute scale), are low-cost, and are thus well adapted to point-of-care scenarios. However, they present several disadvantages for LRS, compared to methods focusing on bacterial cultures: First, they are usually limited to the identification of bacteria present in rather simple matrices, such as urine, while culture-based methods can handle any type of biological sample, including highly complex ones like positive blood cultures. Moreover, these techniques have only been shown to be able to distinguish between biological objects with a significantly different aspect, such as rod-shaped vs. bacillus-shaped bacteria, while our method proposes an identification down to the species level, including cases of true clinical value (e.g., *S. aureus* vs. *S. epidermidis*). Lastly, they are often based on consumables that are complex to manufacture, such as microfluidic chips, while culture-based methods only require Petri dishes, a cheap and easy-to-produce consumable well-adapted to LRS.

Among optical methods focusing on bacterial cultures, colorimetric sensors of microbial volatile metabolites, which yield a fingerprint of the volatile metabolome [36–38] were the first examples of “smart incubators,” i.e., incubators to evaluate the phenotype of growing bacteria continuously, without destroying them or even interrupting the incubation. However, among noninvasive methods of identification, there are still many other aspects of the phenotype to investigate. In particular, optically probing bacteria can evaluate either the chemical composition [39] or the optical phenotype [40].

As far as chemical composition is concerned, vibrational spectroscopies yield a characteristic fingerprint of covalent bonds present in large quantities in biomass, such as C-H or amide or carboxyle bonds [41, 42]. The resulting spectra, analyzed through supervised machine learning, can bring identification down to the species level or even to the strain. As these spectroscopic techniques require the biomass to be probed at different wavelengths, the acquisition of a spectrum may involve complex instrumentation and be time-consuming. Moreover, the high light intensity in use may cause stress to the identified cells, an issue when many colonies are to be analyzed.

These limitations are why probing the optical phenotype is nowadays a serious alternative to spectroscopies. Indeed, optical phenotypes provide information regarding the arrangement of bacteria within colonies, as they are the result of the shape and refractive index of cells, as well as the index and morphotype of the colony itself, including extracellular polymeric substances. This task can be undertaken either through elastic light scattering (ELS) [22, 43] or lensless imaging. ELS allows the colonies of microorganisms to be identified directly on agar without adding reagents by analyzing the scatterogram when targeting a colony with a laser at a submicronic wavelength. ELS requires a strongly coherent light source, i.e., a laser, which only allows the identification of one colony at a time [44]. When a large number of colonies (hundreds or more), have to be identified, ELS is highly time-consuming. Concurrently, lensless imaging uses less coherent lighting, thus allowing for a fast analysis of a large area of the Petri dish.

### 3.2. Paths of Improvement

The analyses presented in Section 2.3 bring out the two most prominent challenges posed by bacterial identification in clinical isolates. First, we have shown that the growth variability of colonies (and, to a lesser extent, morphotypic variants) was the root cause of most of the algorithm's misclassifications. This heavy dependency can be seen not only by parsing the misclassified patches (Section 2.3.1) but is also weaved into the internal representation space of the algorithm (Section 2.3.3). While it's important to note that the accuracies presented in Figure 4 showed that the algorithm has learned partial resilience to this variability, gathering more data to better embrace the diversity of bacterial morphotypes seems unavoidable to further push the misclassification rate down in subsequent studies. To tackle this particular challenge, we plan to exploit a feature of the imaging system that we have not yet put forth in this manuscript: its compactness. Indeed, the imaging system is much less cumbersome than state-of-the-art bacterial identification methods, so much so that it can fit in an incubator. Hence, it is possible to acquire time lapses of Petri dishes as bacteria grow, doing so reveals how much the morphotype of a given colony changes during its growth, in particular regarding the scatterogram (see Figure S3). We expect that including images of bacteria at different growth stages in the training dataset will help the classification algorithm become more robust to the variation of colony size.

The other significant hurdle was the discrimination between closely related species with no obvious distinctive features (Section 2.3.1). Here again, we stress that the relatively high within-order classification accuracy does imply that the algorithm has partially learned to distinguish between such close species pairs. Moreover, the attribution study (Section 2.3.2) highlighted the algorithm's capability to focus on intricate features, be they scatterograms or more subtle characteristics in the rest of the colony, which we hypothesize contain valuable information for species identification. Thus, we believe that classification in further studies could possibly be improved through the acquisition of images at different wavelengths. Indeed, while combining image features acquired at two wavelengths (one in the visible range and another in the infrared one) did not yield a statistically significant gain for this study (see hyperparameter study in section S3.1), we believe such data enrichment to show potential, as the aspect of scatterograms wildly varies across wavelengths [39] (Figure S4). Altogether, we are confident that the coupling of an imaging system capable of generating intricate patterns with an algorithm capable of automatically extracting highly complex features is a promising avenue and that more substantial datasets and improvements in the network's architecture could be sufficient to lead to sizeable performance boosts.

## 4. Conclusion

In this work, we presented a new wide-field lensless imaging system and assessed its capability for bacterial identification. Its large FoV allowed for the creation of a substantial dataset (several tens of thousands of bacterial colonies), which in turn enabled the training of a supervised neural network able to exploit subtle features such as colony scatterograms. This philosophy of acquiring a large amount of raw, rich, visual data and delegating feature engineering to a highly adaptable algorithm is in contrast with commercial state-of-the-art methods such as MALDI-TOF MS, which generate complex physicochemical features, a task both costly and time-consuming. Our work was focused on assessing the true usability of such a technology in field conditions, i.e., on clinical isolates with high colony density. Despite the resulting considerable within-species and within-strain variability in the acquired images, the classification algorithm's performance on the studied species was comparable to state-of-the-art identification methods such as MALDI-TOF MS.

While this study is an important stepping-stone regarding the evaluation of lensless imaging performance for bacterial identification, several next steps are now critical. First, it is crucial to assess the technique's true generalization capability, i.e., the identification performance on *truly* new images outside of our dataset. Indeed, we expect new sources of data variability to arise on several levels, such as specific morphotypic trends of local isolates and variations in the composition of agar and plating technique. Secondly, this study focused on five specific bacterial species, and hence, comparing results with state-of-the-art identification methods capable of recognizing an extensive number of different species is simply indicative. Truly assessing the value of our technique necessarily entails its evaluation on a larger pool of species and in particular the ones of interest in RLS.

To tackle these challenges, we deployed our system in three hospitals in Benin and Burkina Faso via the SIMBLE project, financially supported by the EDCTP [45]. Through these partnerships, we are currently collecting a large dataset covering a wide variety of species prevalent in sub-Saharan Africa. The correct identification of the species in this new dataset constitutes a considerably more ambitious goal than the one achieved in this study, both because of the higher number of species and because of the unavoidable data diversity between the different hospitals. Nevertheless, it will constitute yet a step further in the assessment of this technique's field performance.

## 5. Material and Methods

### 5.1. Imaging System

The lensless imaging system (Figure 1) is composed of an imaging module and an illumination module. The imaging module is adapted from an fp L Interchangeable-lens Mirrorless Type Digital Camera (SIGMA, Aizu, Japan). The image sensor is a full frame (36.0 mm × 24.0 mm) back-illuminated Bayer CMOS sensor, with 62.4 million pixels: an array of 9520 × 6328 pixels at 3.76-*μ*m pitch. We designed and manufactured the electronic system to operate this sensor. The Petri dish is set directly on the sensor and illuminated from above by the illumination module. That module consists of two monochromatic light sources coupled into a multimode optical fiber via a Y fiber SMA (Thorlabs BFY105LS02): a green LED (550 nm) Thorlabs MINTF4 and a near-infrared LED (940 nm) Thorlabs M940F3. An opaque bell-shaped lid is placed on the sensor to guarantee illumination homogeneity across acquisitions. Because the system was deployed in the countries of Sub-Saharan Africa, we carried out a tropicalization procedure by adding a seal to the container and applying a specific polish to protect the electronic card inside. The acquisition of an image requires about 10 s to complete.

### 5.2. Database Acquisition and Data Labelling

Our dataset focused on five bacterial species: two *Enterobacterales* species, *E. coli* and *K. pneumoniae*; two *Staphylococcus* species, *S. aureus* and *S. epidermidis*; and one nonfermentative gram-negative bacillus species, *P. aeruginosa*. *S. epidermidis*, a common contaminant, was included to assess the method's ability to distinguish this species from the closely related pathogen *S. aureus*.

The 252 analyzed isolates of the database were collected at LHUB-ULB in Brussels from positive blood culture, respiratory, and skin/wound and from urine/genital tract samples. All the images were collected on isolates growing on BD Mueller Hinton (MH) II Agar (Becton Dickinson, ref. 254081) after 20 h of growth at 36°C in a conventional Heratherm incubator (Thermo Scientific, ref. 51028130).

As far as blood cultures are concerned, as they are mainly monomicrobial, the imaged MH plates were directly inoculated from positive blood cultures. Only aerobic and pediatric bottles were used, namely BD BACTEC Plus Aerobic/F (Becton Dickinson, ref. 442023) and BD BACTEC Peds Plus/F (Becton Dickinson, ref. 442194). We set a maximum delay of 72 h between positivity and streaking of the MH plate, using a BD Kiestra–automated plate streaking system (Becton Dickinson). We highlight that we willingly chose dilution factors that led to high colony densities, in order to reproduce true field usage of the system. Indeed, when dealing with unknown species, dilution is necessarily species-agnostic and is set such that all tested samples yield a sufficient number of colonies, and consequently, many samples show very high colony densities. This approach is in contrast to other studies based on carefully chosen dilution factors leading to an ideal case of sparse and easily isolated colonies [19, 20, 21].

As far as the other samples are concerned (urine/genital tract, wound/skin, and respiratory specimens), clinical isolates were first grown on an isolation plate, either on MacConkey agar or on a blood-supplemented medium such as Columbia agar. Then, a colony of a specific species, picked from the isolation plate, was resuspended in a suspension broth. Optical density was checked before diluting and finally plating on MH agar. The dilutions of the samples and the volumes used to inoculate the MH plates are summarized in Table 2.

Before inoculating the MH plate, all the studied isolates were identified by the MALDI Biotyper (Bruker): Three clones within a given plate were identified with a minimum score of 2.00 before inoculation on the MH plate (Table S1).

### 5.3. Machine Learning-Based Classification

#### 5.3.1. Data Labelling

Given the task of species identification at the colony scale, several categories of classification algorithms and methods of data labelling could have been implemented. While detection networks could have, in theory, provided identification at the desired scale [20], their training process required the labelling of individual colonies. Such a task was not feasible in our case, as most colonies were so closely regrouped that labelling them individually would have been nonsensical. Even if we had arbitrarily devised borders between clustered colonies, we hypothesized that doing so would have in fact hurt classification performance, as a significant part of the network's capacity would have been devoted to distinguish these senseless borders. Another option consisted in limiting the identification to colonies separated from others, as is often done in studies focusing on laboratory conditions [16, 19, 20, 21, 46]. However, as these isolated colonies represented around 5% of colonies in the dataset (and we had no reason to suppose that this number would be higher in field conditions), such a training method would have considerably reduced the number of individual data points and hence been significantly detrimental to the training process. Moreover, we hypothesized that scatterograms of clustered colonies, while possibly distorted (Figure 3(a)), still held discriminative information regarding the colony species. Hence, we chose to leverage the entirety of the amount of data at hand and to allow for identification in the whole FoV by formalizing this task as a pseudodetection one, in the form of patch classification. Patching is a frequently implemented step in deep learning pipelines applied to biological images [47, 48].

Each image was separated into smaller square subimages (*patches*) of *p* pixel side, nonoverlapping and covering the whole image. Each patch was individually fed to the algorithm, allowing the trained algorithm to produce predictions at that scale (Figure 2, right). Patch classification is less spatially accurate than full-blown object detection, but the output can be of similar clinical value for small patch sizes. For a given patch, we set its associated label to the bacterial species present in the acquisition (all of our samples were monomicrobial) if at least a proportion *c* of the patch was occupied by a colony and to a supplementary category *No colony* otherwise. This latter category was included in the dataset to teach the network to differentiate colonies from the agar gel and possible small debris constituting the background of Petri dishes. The colony occupation of a patch was computed using automatic thresholding followed by a slight human adjustment to prevent markings at the bottom of the Petri dish from being considered colonies.

For all experiments, we set the patch size to *p* = 1000 pixels, meaning that each patch size represented a 3.76-mm wide square region of the full image. This patch size was set to be slightly larger than the typical colony size to allow the algorithm to analyze full scatterograms and small clusters of colonies (see examples of patches in Figures 5, 6, and 7). Thus, images were separated into 54 (6 × 9) patches. We set the minimal colony proportion *c* to 5% of the patch. The number of resulting patches containing colonies was 11,387, roughly balanced over the different species (see Table S2 for the detailed count), and the ones containing no colonies was 2491.

#### 5.3.2. Classification Algorithm

Given that the variability of colony morphotypes was quite high within species, and that the scatterogram features were quite intricate, we explored deep learning methods rather than algorithms based on engineered features. Indeed, the former are known to be much more effective than the latter in computer vision tasks where features are difficult to design manually and when a considerable amount of data is available [49], both conditions being satisfied in this work. Moreover, engineered features are typically manageable to design only for individual colonies (where standard computer vision features such as size and roundness are well-adapted) [19].

In keeping with the state-of-the-art in computer vision tasks in the last decade, we implemented a convolutional neural network to conduct the classification task. A preliminary study was carried out to determine the optimal value of hyperparameters regarding the training procedure and the modalities regarding the data, as thoroughly described in section S3.1. We present here the configuration we consequently adopted in this work. The neural network was a ResNet-18 [50] with no pretraining.

Concerning the training procedure, the batch size was set to 8, the loss was cross-entropy with class balancing, the optimizer was Adam [51] with learning rate of 10^−3^ and weight decay of 5 × 10^−4^. Training was interrupted with early stopping when the loss computed on the annex dataset used for the hyperparameter study did not decrease for eight epochs. Regarding the data itself, basic nondestructive data augmentation was implemented with random flipping and 90° rotation of patches. All of the code was written in Python 3.9 with PyTorch 2.0. The training procedure took about 4 h on an NVIDIA RTX A4000 GPU. We highlight that the chosen neural architecture is several orders of magnitudes smaller than neural networks recently introduced for general purpose computer vision tasks. This decision allowed the analysis phase to be resource efficient, and the prediction of all patches in a single image took less than a second, with no specialized computing unit to accelerate calculations.

#### 5.3.3. Accuracy Computation

An important asset to properly evaluate the applicability of a trained machine learning process is the availability of a *test set* of images, different from the one the network has trained on and representative of the data distribution one can expect in deployment. In our case, regrettably, all images were acquired by the same clinician with the same imaging system in the same laboratory. As we did not have access to a wholly independent dataset to evaluate the algorithm (e.g., acquired in a different hospital), we simulated an independent test set by implementing *k*-fold cross-validation on our dataset, as it is usually done to evaluate machine learning pipeline performances with no access to a true test set. We set *k* to 5. We emphasize that the assignment of patches to folds was carried out at the full image level, meaning that *all* patches from a given image were either in a fold used for training or the fold used for testing. Hence, albeit limited to a single clinical setting, the test set did evaluate the generalization of the algorithm to new clinical isolates.

Interestingly, the standard deviation of accuracy across cross-validation passes was somewhat high regarding species identification. For instance, the accuracy difference for species between the worst and the best passes was 5.5%. Closer inspection revealed that these differences in accuracy were mostly caused by variations in the proportion of strongly atypical morphotypes in the test fold. While seemingly discouraging, this variation highlights the significant impact of such morphotypes on classification performance. This result further strengthened our conviction that evaluating an algorithm with a cross-validation scheme on clinical data leads to a closer estimation of its actual deployment accuracy than the one reference strain studies can offer. All post hoc analyses presented in Section 2.3 were conducted with the network trained during the cross-validation pass that had led to the best accuracy.

## Figures and Tables

**Table 1:** Number of images acquired for each species and each specimen type.

	**Blood**	**Urine**	**Skin**	**Respiratory**	**Total**
*Escherichia coli* (*E. coli*)	18	15	11	13	58
*Klebsiella pneumoniae* (*K. pneumoniae*)	16	15	6	11	47
*Pseudomonas aeruginosa* (*P. aeruginosa*)	12	17	10	16	55
*Staphylococcus aureus* (*S. aureus*)	21	6	10	10	47
*Staphylococcus epidermidis* (*S. epidermidis*)	19	8	9	9	45
Total	86	61	46	59	252

**Table 2:** Inoculation and dilution conditions for each species.

	**Blood**	**Urine**	**Skin**	**Respiratory**
*E. coli*	10 *μ*L (1:75)	10 *μ*L (1:5–McF 0.5)	10 *μ*L (1:5–McF 0.5)	10 *μ*L (1:5–McF 0.5)
*K. pneumoniae*	10 *μ*L (1:75)	10 *μ*L (1:10–McF 0.5)	10 *μ*L (1:5–McF 0.5)	10 *μ*L (1:5–McF 0.5)
*P. aeruginosa*	10 *μ*L (1:75)	10 *μ*L (1:20–McF 0.5)	10 *μ*L (1:20–McF 0.5)	10 *μ*L (1:20–McF 0.5)
*S. aureus*	10 *μ*L (1:10)	10 *μ*L (pure–McF 0.5)	10 *μ*L (pure–McF 0.5)	10 *μ*L (pure–McF 0.5)
*S. epidermidis*	10 *μ*L (1:10)	10 *μ*L (pure–McF 0.5)	10 *μ*L (pure–McF 0.5)	10 *μ*L (pure–McF 0.5)

## Data Availability

The images of bacterial colonies that we acquired for this study are freely available online [53] (10.6019/S-BIAD1096). The dataset includes all 252 acquired images as well as binary annotation masks indicating the presence/absence of colonies in each image.

## Appendix A: Code Availability

The trained neural network described in this study is freely available online [52] (10.5281/zenodo.10931149) along with a script to carry out inference with user-provided images.

## Supporting Information

Supporting Information Additional supporting information can be found online in the Supporting Information section. A supporting information file is associated with this manuscript. This file contains the following items, all referenced in the main text. Figure S1: phylogenetic tree of the studied species. Figure S2: several images acquired with the imaging system. Figure S3: timelapses of two colonies over 6 h. Figure S4: comparison of the morphotypes of colonies illuminated with different wavelengths. Figure S5: hyperparameter optimization results. Figure S6: several images suffering from patching artifacts. Table S1: MALDI-TOF MS identification scores for the clinical isolates of this study. Table S2: breakdown of the number of patches in the dataset used to train the neural network. Table S3: list of studied hyperparameters and their tested values during the hyperparameter optimization process.
