# Supplementary material for: Simple Imaging System for Label-Free Identification of Bacterial Pathogens in Resource-Limited Settings
Source: Int J Biomed Imaging. 2024 Nov 19;2024:6465280. doi: 10.1155/2024/6465280 (PMC11599477; doi:10.1155/2024/6465280)
Supplement: Supporting Information — Additional supporting information can be found online in the Supporting Information section. A supporting information file is associated with this manuscript. This file contains the following items, all referenced in the main text. Figure S1: phylogenetic tree of the studied species. Figure S2: several images acquired with the imaging system. Figure S3: timelapses of two colonies over 6 h. Figure S4: comparison of the morphotypes of colonies illuminated with different wavelengths. Figure S5: hyperparameter optimization results. Figure S6: several images suffering from patching artifacts. Table S1: MALDI-TOF MS identification scores for the clinical isolates of this study. Table S2: breakdown of the number of patches in the dataset used to train the neural network. Table S3: list of studied hyperparameters and their tested values during the hyperparameter optimization process. [file 6465280.f1.pdf]

# Supplementary material

## 1. Characterisation of clinical isolates

| Sample ID | Specimen type | MALDI-TOF ID | Score 1 | Score 2 | Score 3 |
|---|---|---|---|---|---|
| U16937711BEC | blood culture | *E. coli* | 2.24 | 2.18 | 2.15 |
| U25348020BEC | blood culture | *E. coli* | 2.23 | 2.22 | 2.21 |
| U25349406BEC | blood culture | *E. coli* | 2.35 | 2.31 | 2.30 |
| U25353696BEC | blood culture | *E. coli* | 2.44 | 2.43 | 2.36 |
| U25355503BEC | blood culture | *E. coli* | 2.52 | 2.47 | 2.38 |
| U25368462BEC | blood culture | *E. coli* | 2.30 | 2.28 | 2.22 |
| U25369847BEC | blood culture | *E. coli* | 2.27 | 2.18 | 2.07 |
| U25371090BEC | blood culture | *E. coli* | 2.32 | 2.23 | 2.21 |
| U25372465BEC | blood culture | *E. coli* | 2.22 | 2.18 | 2.13 |
| U25372754BEC | blood culture | *E. coli* | 2.35 | 2.26 | 2.21 |
| U29764260BEC | blood culture | *E. coli* | 2.22 | 2.18 | 2.17 |
| U31311704BEC | blood culture | *E. coli* | 2.41 | 2.40 | 2.31 |
| U31313591BEC | blood culture | *E. coli* | 2.22 | 2.05 | 2.01 |
| U48716421BEC | blood culture | *E. coli* | 2.40 | 2.29 | 2.24 |
| U48728237BEC | blood culture | *E. coli* | 2.21 | 2.08 | 2.08 |
| U48728406BEC | blood culture | *E. coli* | 2.34 | 2.31 | 2.29 |
| U48730687BEC | blood culture | *E. coli* | 2.38 | 2.34 | 2.31 |
| U48730727BEC | blood culture | *E. coli* | 2.34 | 2.19 | 2.14 |
| U81653530BEC | blood culture | *E. coli* | 2.28 | 2.20 | 2.17 |
| U25355915BKP | blood culture | *K. pneumoniae* | 2.36 | 2.30 | 2.11 |
| U25381065BKP | blood culture | *K. pneumoniae* | 2.44 | 1.66 | 1.60 |
| U31307909BKP | blood culture | *K. pneumoniae* | 2.17 | 2.04 | 1.89 |
| U31310233BKP | blood culture | *K. pneumoniae* | 2.33 | 2.21 | 2.20 |
| U31317128BKP | blood culture | *K. pneumoniae* | 2.39 | 2.28 | 2.20 |
| U31334799BKP | blood culture | *K. pneumoniae* | 2.18 | 2.17 | 2.09 |
| U31338592BKP | blood culture | *K. pneumoniae* | 2.48 | 2.42 | 2.35 |
| U362XYJBKP | blood culture | *K. pneumoniae* | 2.11 | 2.07 | 2.03 |
| U48688640BKP | blood culture | *K. pneumoniae* | 2.47 | 2.44 | 2.41 |
| U48689215BKP | blood culture | *K. pneumoniae* | 2.41 | 2.29 | 2.19 |
| U48692452BKP | blood culture | *K. pneumoniae* | 2.24 | 2.13 | 2.05 |
| U48727327BKP | blood culture | *K. pneumoniae* | 2.39 | 2.25 | 2.08 |
| U48731576BKP | blood culture | *K. pneumoniae* | 2.19 | 2.19 | 2.15 |
| UO77ACKBKP | blood culture | *K. pneumoniae* | 2.48 | 2.36 | 2.30 |
| UO78ACKBKP | blood culture | *K. pneumoniae* | 2.20 | 2.01 | 2.00 |
| U16740618BPA | blood culture | *P. aeruginosa* | 2.34 | 2.25 | 2.18 |
| U25355503BPA | blood culture | *P. aeruginosa* | 2.29 | 2.17 | 2.13 |
| U31313379BPA | blood culture | *P. aeruginosa* | 2.32 | 2.29 | 2.22 |
| U31316559BPA | blood culture | *P. aeruginosa* | 2.16 | 2.11 | 2.00 |
| U48712749BPA | blood culture | *P. aeruginosa* | 2.33 | 2.13 | 2.09 |
| U48718229BPA | blood culture | *P. aeruginosa* | 2.13 | 2.07 | 2.06 |

| | | | | | |
|---|---|---|---|---|---|
| U48718401BPA | blood culture | *P. aeruginosa* | 2.06 | 1.69 | 1.60 |
| U48722242BPA | blood culture | *P. aeruginosa* | 2.42 | 2.15 | 2.12 |
| U48728323BPA | blood culture | *P. aeruginosa* | 2.29 | 2.22 | 2.18 |
| U48733302BPA | blood culture | *P. aeruginosa* | 2.45 | 2.37 | 2.15 |
| U807XSJBPA | blood culture | *P. aeruginosa* | 2.43 | 2.29 | 2.20 |
| U81663716BPA | blood culture | *P. aeruginosa* | 2.12 | 2.01 | 2.01 |
| U16742978BSA | blood culture | *S. aureus* | 2.39 | 2.38 | 2.36 |
| U16743813BSA | blood culture | *S. aureus* | 2.31 | 2.26 | 2.18 |
| U16743822BSA | blood culture | *S. aureus* | 2.42 | 2.36 | 2.30 |
| U25343206BSA | blood culture | *S. aureus* | 2.40 | 2.37 | 2.36 |
| U25345000BSA | blood culture | *S. aureus* | 2.30 | 2.30 | 2.20 |
| U25356071BSA | blood culture | *S. aureus* | 2.28 | 2.20 | 2.20 |
| U25365353BSA | blood culture | *S. aureus* | 2.27 | 2.17 | 2.14 |
| U25367212BSA | blood culture | *S. aureus* | 2.17 | 2.15 | 2.11 |
| U25420608BSA | blood culture | *S. aureus* | 2.26 | 2.24 | 2.19 |
| U25421239BSA | blood culture | *S. aureus* | 2.39 | 2.35 | 2.35 |
| U25422000BSA | blood culture | *S. aureus* | 2.37 | 2.36 | 2.23 |
| U25426845BSA | blood culture | *S. aureus* | 2.41 | 2.37 | 2.35 |
| U29642626BSA | blood culture | *S. aureus* | 2.39 | 2.36 | 2.29 |
| U29738955BSA | blood culture | *S. aureus* | 2.20 | 2.13 | 2.04 |
| U29766208BSA | blood culture | *S. aureus* | 2.25 | 2.22 | 2.20 |
| U31301173BSA | blood culture | *S. aureus* | 2.25 | 2.21 | 2.19 |
| U48689742BSA | blood culture | *S. aureus* | 2.29 | 2.29 | 2.29 |
| U48728059BSA | blood culture | *S. aureus* | 2.42 | 2.34 | 2.33 |
| U48731039BSA | blood culture | *S. aureus* | 2.34 | 2.22 | 2.15 |
| U48731406BSA | blood culture | *S. aureus* | 2.40 | 2.39 | 2.30 |
| U81664631BSA | blood culture | *S. aureus* | 2.30 | 2.28 | 2.22 |
| U16765478BSE | blood culture | *S. epidermidis* | 2.19 | 2.14 | 2.08 |
| U17103748BSE | blood culture | *S. epidermidis* | 2.20 | 2.16 | 2.14 |
| U25344268BSE | blood culture | *S. epidermidis* | 2.20 | 1.95 | 1.93 |
| U25354589BSE | blood culture | *S. epidermidis* | 2.21 | 2.13 | 2.11 |
| U25355288BSE | blood culture | *S. epidermidis* | 2.04 | 2.02 | 2.01 |
| U25367978BSE | blood culture | *S. epidermidis* | 2.10 | 2.08 | 1.97 |
| U25371787BSE | blood culture | *S. epidermidis* | 2.17 | 2.17 | 2.13 |
| U25376437BSE | blood culture | *S. epidermidis* | 2.04 | 1.98 | 1.92 |
| U31297485BSE | blood culture | *S. epidermidis* | 2.07 | 1.80 | 1.67 |
| U31300893BSE | blood culture | *S. epidermidis* | 2.04 | 2.01 | 1.86 |
| U31302533BSE | blood culture | *S. epidermidis* | 2.26 | 2.19 | 2.13 |
| U31317246BSE | blood culture | *S. epidermidis* | 2.02 | 1.92 | 1.89 |
| U48715542BSE | blood culture | *S. epidermidis* | 2.17 | 2.15 | 2.12 |
| U48728397BSE | blood culture | *S. epidermidis* | 2.23 | 2.13 | 2.10 |
| U81649037BSE | blood culture | *S. epidermidis* | 2.29 | 2.26 | 2.13 |
| U81649056BSE | blood culture | *S. epidermidis* | 2.15 | 1.98 | 1.98 |
| U81652262BSE | blood culture | *S. epidermidis* | 2.20 | 2.18 | 2.14 |
| U89271349BSE | blood culture | *S. epidermidis* | 2.26 | 2.12 | 2.08 |
| U25374206BSE | blood culture | *S. epidermidis* | 2.27 | 2.13 | 2.11 |

| | | | | | |
|---|---|---|---|---|---|
| U16742557REC | respiratory | *E. coli* | 2.37 | 2.34 | 2.33 |
| U25353378REC | respiratory | *E. coli* | 2.41 | 2.28 | 2.22 |
| U25356386REC | respiratory | *E. coli* | 2.41 | 2.35 | 2.31 |
| U25436616REC | respiratory | *E. coli* | 2.36 | 2.34 | 2.32 |
| U25438434REC | respiratory | *E. coli* | 2.37 | 2.27 | 2.23 |
| U25441048REC | respiratory | *E. coli* | 2.46 | 2.35 | 2.31 |
| U25460488REC | respiratory | *E. coli* | 2.45 | 2.42 | 2.32 |
| U31341701REC | respiratory | *E. coli* | 2.44 | 2.39 | 2.35 |
| U31344297REC | respiratory | *E. coli* | 2.36 | 2.35 | 2.28 |
| U31351004REC | respiratory | *E. coli* | 2.40 | 2.39 | 2.32 |
| U48682656REC | respiratory | *E. coli* | 2.48 | 2.43 | 2.40 |
| U81649301REC | respiratory | *E. coli* | 2.24 | 2.20 | 2.15 |
| U25357992RKP | respiratory | *K. pneumoniae* | 2.47 | 2.42 | 2.31 |
| U25426316RKP | respiratory | *K. pneumoniae* | 2.21 | 2.16 | 2.12 |
| U25428738RKP | respiratory | *K. pneumoniae* | 2.53 | 2.44 | 2.44 |
| U25429602RKP | respiratory | *K. pneumoniae* | 2.35 | 2.35 | 2.19 |
| U25439342RKP | respiratory | *K. pneumoniae* | 2.47 | 2.37 | 2.36 |
| U25440392RKP | respiratory | *K. pneumoniae* | 2.49 | 2.42 | 2.38 |
| U25454013RKP | respiratory | *K. pneumoniae* | 2.47 | 2.34 | 2.33 |
| U25457857RKP | respiratory | *K. pneumoniae* | 2.32 | 2.30 | 2.16 |
| U29771657RKP | respiratory | *K. pneumoniae* | 2.47 | 2.37 | 2.35 |
| U31337957RKP | respiratory | *K. pneumoniae* | 2.31 | 2.18 | 2.11 |
| U31339726RKP | respiratory | *K. pneumoniae* | 2.34 | 2.15 | 2.14 |
| U11991085RPA | respiratory | *P. aeruginosa* | 2.38 | 2.30 | 2.29 |
| U11992759RPA | respiratory | *P. aeruginosa* | 2.39 | 2.38 | 2.28 |
| U11993880RPA | respiratory | *P. aeruginosa* | 2.35 | 2.29 | 2.22 |
| U16765046RPA | respiratory | *P. aeruginosa* | 2.34 | 2.29 | 2.28 |
| U16816314RPA | respiratory | *P. aeruginosa* | 2.15 | 2.09 | 2.08 |
| U25353088RPA | respiratory | *P. aeruginosa* | 2.37 | 2.36 | 2.35 |
| U25353913RPA | respiratory | *P. aeruginosa* | 2.44 | 2.26 | 2.25 |
| U25354112RPA | respiratory | *P. aeruginosa* | 2.36 | 2.29 | 2.28 |
| U25354729RPA | respiratory | *P. aeruginosa* | 2.32 | 2.29 | 2.26 |
| U25354908RPA | respiratory | *P. aeruginosa* | 2.35 | 2.23 | 2.19 |
| U253S4908RPA | respiratory | *P. aeruginosa* | 2.16 | 1.97 | 1.85 |
| U48728281RPA | respiratory | *P. aeruginosa* | 2.39 | 2.33 | 2.30 |
| U81648159RPA | respiratory | *P. aeruginosa* | 2.31 | 2.23 | 2.22 |
| U81648160RPA | respiratory | *P. aeruginosa* | 2.34 | 2.27 | 2.23 |
| U81648476RPA | respiratory | *P. aeruginosa* | 2.30 | 2.27 | 2.20 |
| UI1993880RPA | respiratory | *P. aeruginosa* | 2.38 | 2.35 | 2.30 |
| U11992386RSA | respiratory | *S. aureus* | 2.33 | 2.33 | 2.30 |
| U11993216RSA | respiratory | *S. aureus* | 2.35 | 2.34 | 2.32 |
| U25353186RSA | respiratory | *S. aureus* | 2.45 | 2.38 | 2.37 |
| U25353502RSA | respiratory | *S. aureus* | 2.31 | 2.29 | 2.28 |
| U25353913RSA | respiratory | *S. aureus* | 2.42 | 2.37 | 2.35 |
| U31301810RSA | respiratory | *S. aureus* | 2.40 | 2.38 | 2.34 |
| U81644818RSA | respiratory | *S. aureus* | 2.29 | 2.25 | 2.15 |

| | | | | | |
|---|---|---|---|---|---|
| U81648136RSA | respiratory | *S. aureus* | 2.39 | 2.36 | 2.36 |
| U81648507RSA | respiratory | *S. aureus* | 2.44 | 2.38 | 2.31 |
| U89254921RSA | respiratory | *S. aureus* | 2.33 | 2.32 | 2.32 |
| U16929206RSE | respiratory | *S. epidermidis* | 2.24 | 2.24 | 2.21 |
| U25356080RSE | respiratory | *S. epidermidis* | 2.17 | 2.10 | 2.08 |
| U31345333RSE | respiratory | *S. epidermidis* | 2.03 | 1.97 | 1.89 |
| U31345754RSE | respiratory | *S. epidermidis* | 2.24 | 2.20 | 2.07 |
| U31349408RSE | respiratory | *S. epidermidis* | 2.23 | 2.12 | 2.04 |
| U31352606RSE | respiratory | *S. epidermidis* | 2.11 | 2.08 | 2.07 |
| U48674826RSE | respiratory | *S. epidermidis* | 2.23 | 2.07 | 2.07 |
| U48687575RSE | respiratory | *S. epidermidis* | 2.25 | 2.04 | 2.04 |
| U48727550RSE | respiratory | *S. epidermidis* | 2.20 | 2.17 | 2.06 |
| U16765061SEC | skin / wound | *E. coli* | 2.30 | 2.23 | 2.23 |
| U16765749SEC | skin / wound | *E. coli* | 2.40 | 2.38 | 2.36 |
| U16937400SEC | skin / wound | *E. coli* | 2.19 | 2.15 | 2.13 |
| U25356129SEC | skin / wound | *E. coli* | 2.41 | 2.35 | 2.34 |
| U25356133SEC | skin / wound | *E. coli* | 2.16 | 2.14 | 2.06 |
| U25356148SEC | skin / wound | *E. coli* | 2.40 | 2.36 | 2.31 |
| U31302047SEC | skin / wound | *E. coli* | 2.43 | 2.39 | 2.38 |
| U48722064SEC | skin / wound | *E. coli* | 2.24 | 2.16 | 2.16 |
| U48728001SEC | skin / wound | *E. coli* | 2.32 | 2.27 | 2.27 |
| U48728529SEC | skin / wound | *E. coli* | 2.28 | 2.27 | 2.26 |
| U48729338SEC | skin / wound | *E. coli* | 2.29 | 2.26 | 2.22 |
| UI6765061SEC | skin / wound | *E. coli* | 2.15 | 2.14 | 2.10 |
| U25366065SKP | skin / wound | *K. pneumoniae* | 2.26 | 2.21 | 2.16 |
| U25366097SKP | skin / wound | *K. pneumoniae* | 2.32 | 2.31 | 2.22 |
| U31302405SKP | skin / wound | *K. pneumoniae* | 2.48 | 2.45 | 2.36 |
| U31303293SKP | skin / wound | *K. pneumoniae* | 2.36 | 2.25 | 2.22 |
| U48722087SKP | skin / wound | *K. pneumoniae* | 2.22 | 2.21 | 2.09 |
| U48727741SKP | skin / wound | *K. pneumoniae* | 2.33 | 2.30 | 2.20 |
| U16765216SPA | skin / wound | *P. aeruginosa* | 2.17 | 2.08 | 1.95 |
| U16771077SPA | skin / wound | *P. aeruginosa* | 2.50 | 2.25 | 2.24 |
| U16842786SPA | skin / wound | *P. aeruginosa* | 2.27 | 2.25 | 2.23 |
| U25353510SPA | skin / wound | *P. aeruginosa* | 2.28 | 2.12 | 2.11 |
| U25355375SPA | skin / wound | *P. aeruginosa* | 2.35 | 2.23 | 2.16 |
| U25362962SPA | skin / wound | *P. aeruginosa* | 2.21 | 2.13 | 2.11 |
| U31302454SPA | skin / wound | *P. aeruginosa* | 2.33 | 2.31 | 2.28 |
| U48724132SPA | skin / wound | *P. aeruginosa* | 2.35 | 2.33 | 2.32 |
| U48728400SPA | skin / wound | *P. aeruginosa* | 2.33 | 2.29 | 2.28 |
| U48728506SPA | skin / wound | *P. aeruginosa* | 2.22 | 2.17 | 2.10 |
| U25353587SSA | skin / wound | *S. aureus* | 2.16 | 2.11 | 2.06 |
| U25354089SSA | skin / wound | *S. aureus* | 2.34 | 2.32 | 2.31 |
| U25354968SSA | skin / wound | *S. aureus* | 2.35 | 2.35 | 2.35 |
| U25355292SSA | skin / wound | *S. aureus* | 2.31 | 2.29 | 2.26 |
| U31302454SSA | skin / wound | *S. aureus* | 2.44 | 2.42 | 2.40 |
| U31302546SSA | skin / wound | *S. aureus* | 2.38 | 2.37 | 2.37 |

| | | | | | |
|---|---|---|---|---|---|
| U31302729SSA | skin / wound | *S. aureus* | 2.23 | 2.19 | 2.16 |
| U31303470SSA | skin / wound | *S. aureus* | 2.25 | 2.12 | 2.10 |
| U48730072SSA | skin / wound | *S. aureus* | 2.41 | 2.38 | 2.33 |
| U81648871SSA | skin / wound | *S. aureus* | 2.30 | 2.29 | 2.24 |
| U16765338SSE | skin / wound | *S. epidermidis* | 2.22 | 2.13 | 2.09 |
| U25354246SSE | skin / wound | *S. epidermidis* | 2.23 | 2.13 | 2.04 |
| U31303575SSE | skin / wound | *S. epidermidis* | 2.19 | 2.15 | 2.04 |
| U48723234SSE | skin / wound | *S. epidermidis* | 2.13 | 2.07 | 1.97 |
| U48727343SSE | skin / wound | *S. epidermidis* | 2.10 | 2.00 | 1.88 |
| U48727550SSE | skin / wound | *S. epidermidis* | 2.17 | 2.13 | 2.08 |
| U48728328SSE | skin / wound | *S. epidermidis* | 2.29 | 2.25 | 2.24 |
| U48728830SSE | skin / wound | *S. epidermidis* | 2.10 | 2.10 | 2.02 |
| UI6765338SSE | skin / wound | *S. epidermidis* | 2.03 | 2.03 | 2.01 |
| U25353282UEC | urine / genital tract | *E. coli* | 2.52 | 2.34 | 2.33 |
| U25354062UEC | urine / genital tract | *E. coli* | 2.34 | 2.32 | 2.25 |
| U25354352UEC | urine / genital tract | *E. coli* | 2.33 | 2.27 | 2.22 |
| U25354782UEC | urine / genital tract | *E. coli* | 2.37 | 2.30 | 2.29 |
| U25366308UEC | urine / genital tract | *E. coli* | 2.09 | 2.06 | 2.02 |
| U25366569UEC | urine / genital tract | *E. coli* | 2.25 | 2.19 | 2.12 |
| U29756714UEC | urine / genital tract | *E. coli* | 2.38 | 2.34 | 1.68 |
| U29759367UEC | urine / genital tract | *E. coli* | 2.40 | 2.33 | 2.30 |
| U31301976UEC | urine / genital tract | *E. coli* | 2.21 | 2.09 | 2.07 |
| U31302442UEC | urine / genital tract | *E. coli* | 2.30 | 2.26 | 2.24 |
| U31302678UEC | urine / genital tract | *E. coli* | 2.37 | 2.32 | 2.29 |
| U31307772UEC | urine / genital tract | *E. coli* | 2.44 | 2.34 | 2.33 |
| U31308479UEC | urine / genital tract | *E. coli* | 2.19 | 2.15 | 2.13 |
| U31308488UEC | urine / genital tract | *E. coli* | 2.21 | 2.16 | 2.14 |
| U81648964UEC | urine / genital tract | *E. coli* | 2.29 | 2.29 | 2.16 |
| U25353291UKP | urine / genital tract | *K. pneumoniae* | 2.41 | 2.40 | 2.34 |
| U25354048UKP | urine / genital tract | *K. pneumoniae* | 2.31 | 2.29 | 2.23 |
| U25354225UKP | urine / genital tract | *K. pneumoniae* | 2.24 | 2.17 | 2.16 |
| U25356285UKP | urine / genital tract | *K. pneumoniae* | 2.51 | 2.40 | 2.40 |
| U25356333UKP | urine / genital tract | *K. pneumoniae* | 2.43 | 2.31 | 2.30 |
| U25357552UKP | urine / genital tract | *K. pneumoniae* | 2.35 | 2.35 | 2.32 |
| U25364609UKP | urine / genital tract | *K. pneumoniae* | 2.46 | 2.41 | 2.32 |
| U31301262UKP | urine / genital tract | *K. pneumoniae* | 2.36 | 2.36 | 2.29 |
| U31303332UKP | urine / genital tract | *K. pneumoniae* | 2.39 | 2.38 | 2.28 |
| U48722690UKP | urine / genital tract | *K. pneumoniae* | 2.39 | 2.26 | 2.21 |
| U48726580UKP | urine / genital tract | *K. pneumoniae* | 2.40 | 2.38 | 2.33 |
| U48727991UKP | urine / genital tract | *K. pneumoniae* | 2.49 | 2.38 | 2.36 |
| U81649045UKP | urine / genital tract | *K. pneumoniae* | 2.23 | 2.22 | 2.20 |
| U81649140UKP | urine / genital tract | *K. pneumoniae* | 2.47 | 2.44 | 2.42 |
| U81649157UKP | urine / genital tract | *K. pneumoniae* | 2.42 | 2.33 | 2.31 |
| U25353258UPA | urine / genital tract | *P. aeruginosa* | 2.17 | 2.11 | 2.04 |
| U25353498UPA | urine / genital tract | *P. aeruginosa* | 2.23 | 2.20 | 2.13 |
| U25356423UPA | urine / genital tract | *P. aeruginosa* | 2.21 | 2.14 | 2.05 |

| | | | | | |
|---|---|---|---|---|---|
| U25356465UPA | urine / genital tract | *P. aeruginosa* | 2.39 | 2.36 | 2.33 |
| U25363351UPA | urine / genital tract | *P. aeruginosa* | 2.05 | 2.03 | 2.00 |
| U25364126UPA | urine / genital tract | *P. aeruginosa* | 2.40 | 2.39 | 2.33 |
| U25365665UPA | urine / genital tract | *P. aeruginosa* | 2.29 | 2.22 | 2.18 |
| U25366074UPA | urine / genital tract | *P. aeruginosa* | 2.28 | 2.24 | 2.14 |
| U253S3258UPA | urine / genital tract | *P. aeruginosa* | 2.20 | 2.09 | 2.09 |
| U2S356423UPA | urine / genital tract | *P. aeruginosa* | 2.30 | 2.20 | 2.09 |
| U31303264UPA | urine / genital tract | *P. aeruginosa* | 2.37 | 2.16 | 2.14 |
| U31303412UPA | urine / genital tract | *P. aeruginosa* | 2.41 | 2.29 | 2.25 |
| U31303434UPA | urine / genital tract | *P. aeruginosa* | 2.26 | 2.24 | 2.22 |
| U31308221UPA | urine / genital tract | *P. aeruginosa* | 2.27 | 2.21 | 2.17 |
| U48722361UPA | urine / genital tract | *P. aeruginosa* | 2.27 | 2.22 | 2.14 |
| U48727119UPA | urine / genital tract | *P. aeruginosa* | 2.37 | 2.23 | 2.16 |
| U81648941UPA | urine / genital tract | *P. aeruginosa* | 2.42 | 2.23 | 2.21 |
| U12032399USA | urine / genital tract | *S. aureus* | 2.42 | 2.33 | 2.32 |
| U12032800USA | urine / genital tract | *S. aureus* | 2.45 | 2.36 | 2.35 |
| U16762365USA | urine / genital tract | *S. aureus* | 2.28 | 2.20 | 2.16 |
| U25356609USA | urine / genital tract | *S. aureus* | 2.43 | 2.35 | 2.34 |
| U25364819USA | urine / genital tract | *S. aureus* | 2.30 | 2.24 | 2.23 |
| U48730275USA | urine / genital tract | *S. aureus* | 2.43 | 2.41 | 2.37 |
| U10000000USE | urine / genital tract | *S. epidermidis* | 2.24 | 2.18 | 2.11 |
| U25352104USE | urine / genital tract | *S. epidermidis* | 2.16 | 2.16 | 2.13 |
| U25354842USE | urine / genital tract | *S. epidermidis* | 2.24 | 2.22 | 2.15 |
| U25357347USE | urine / genital tract | *S. epidermidis* | 2.10 | 2.06 | 2.03 |
| U25429422USE | urine / genital tract | *S. epidermidis* | 2.09 | 2.06 | 2.00 |
| U25430707USE | urine / genital tract | *S. epidermidis* | 2.35 | 2.16 | 2.12 |
| U48685944USE | urine / genital tract | *S. epidermidis* | 2.27 | 2.16 | 2.08 |
| U48726825USE | urine / genital tract | *S. epidermidis* | 2.00 | 1.87 | 1.72 |

*Table S1 : MALDI-TOF MS identification scores for the clinical isolates of this study. Scores are highlighted depending on their value: green for scores above 2.0 (included) (high confidence identification), yellow for scores between 1.7 (included) and 2.0 (excluded) (low confidence identification), red for scores under 1.7 (excluded) (no organism identification possible).*

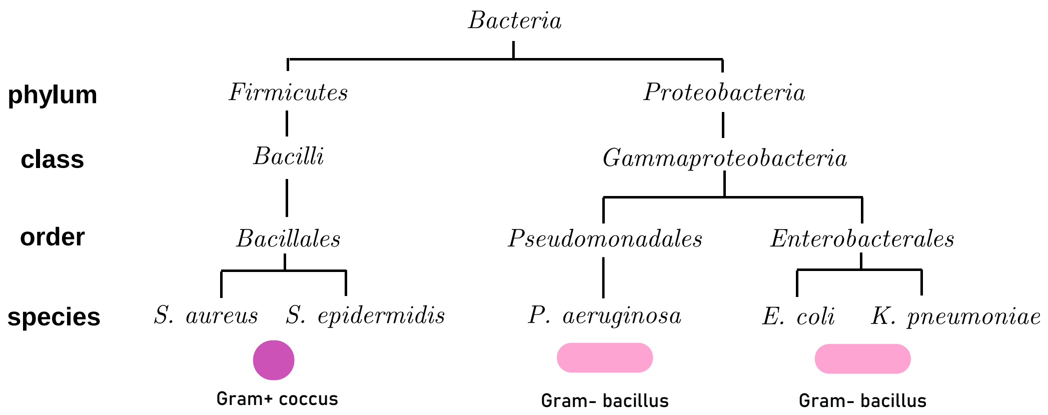


*Figure S1 : Phylogenetic tree of the studied species.*

# 2. Dataset

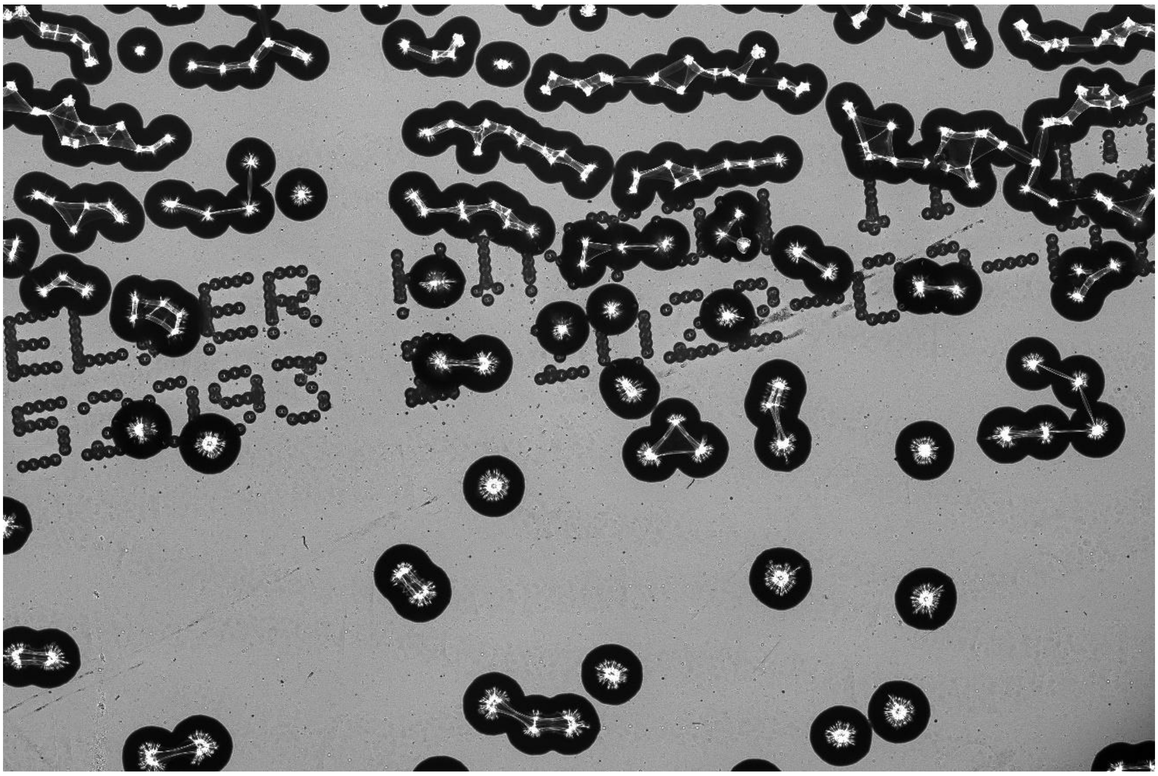

(a)

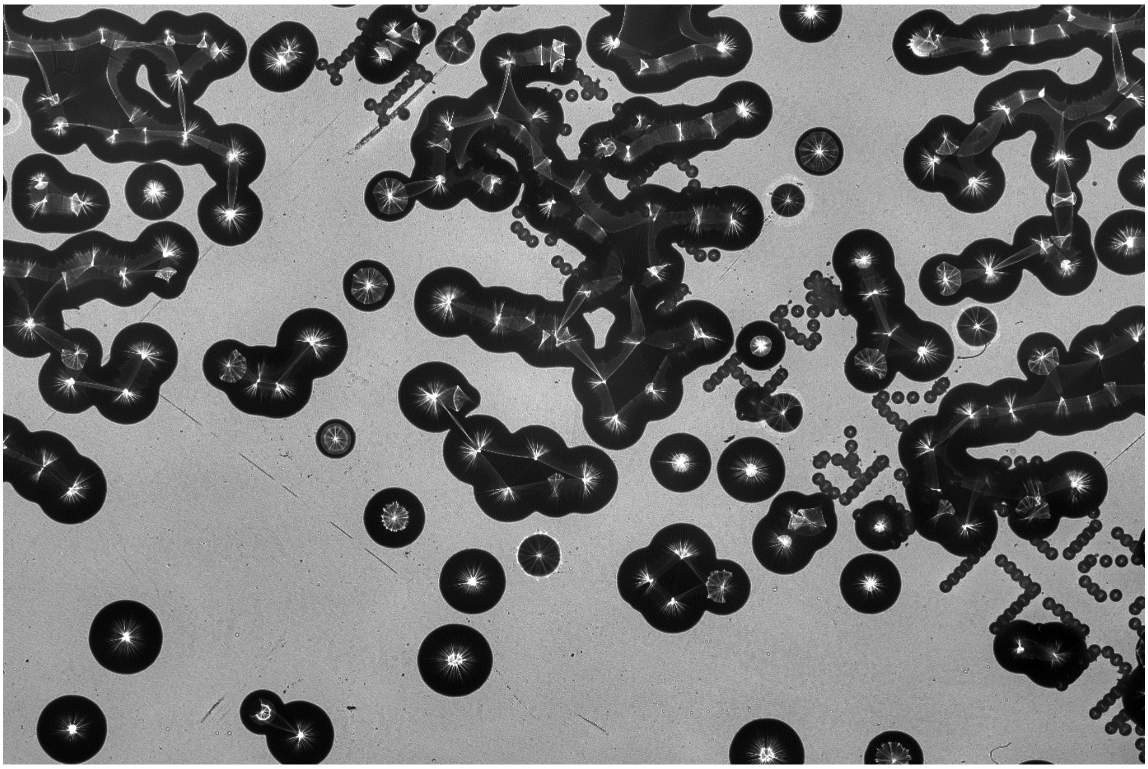

(b)

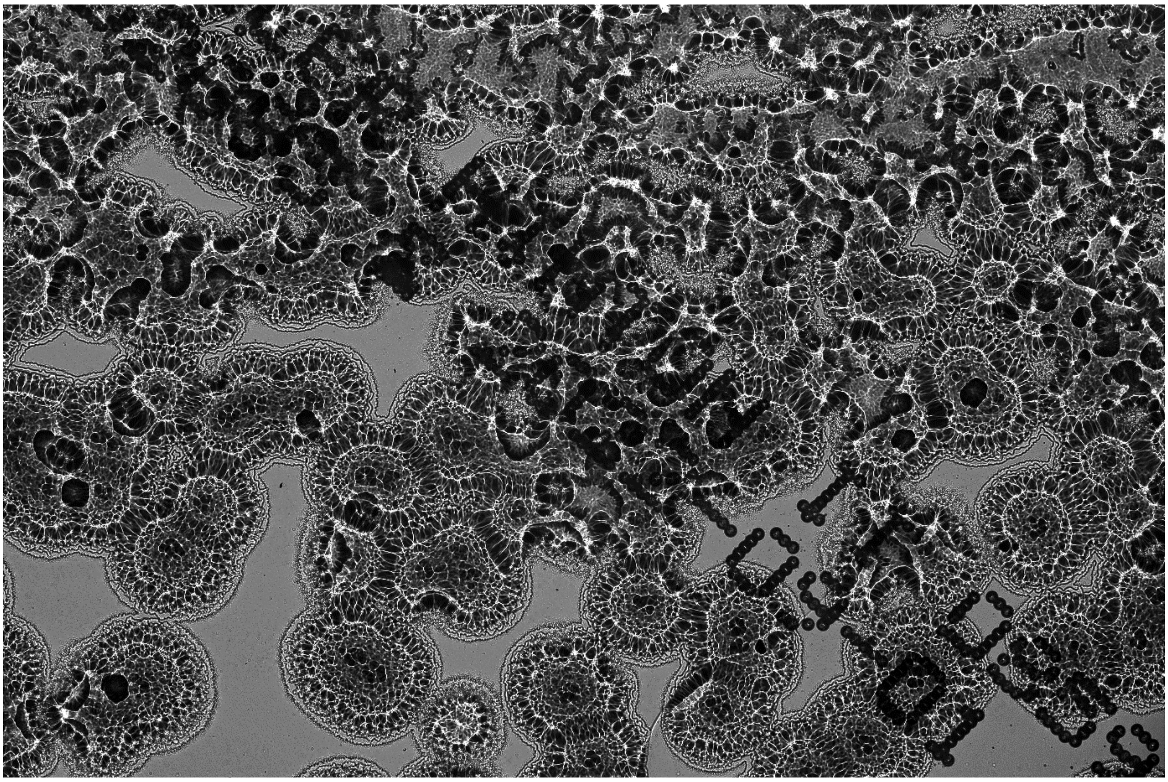

(c)

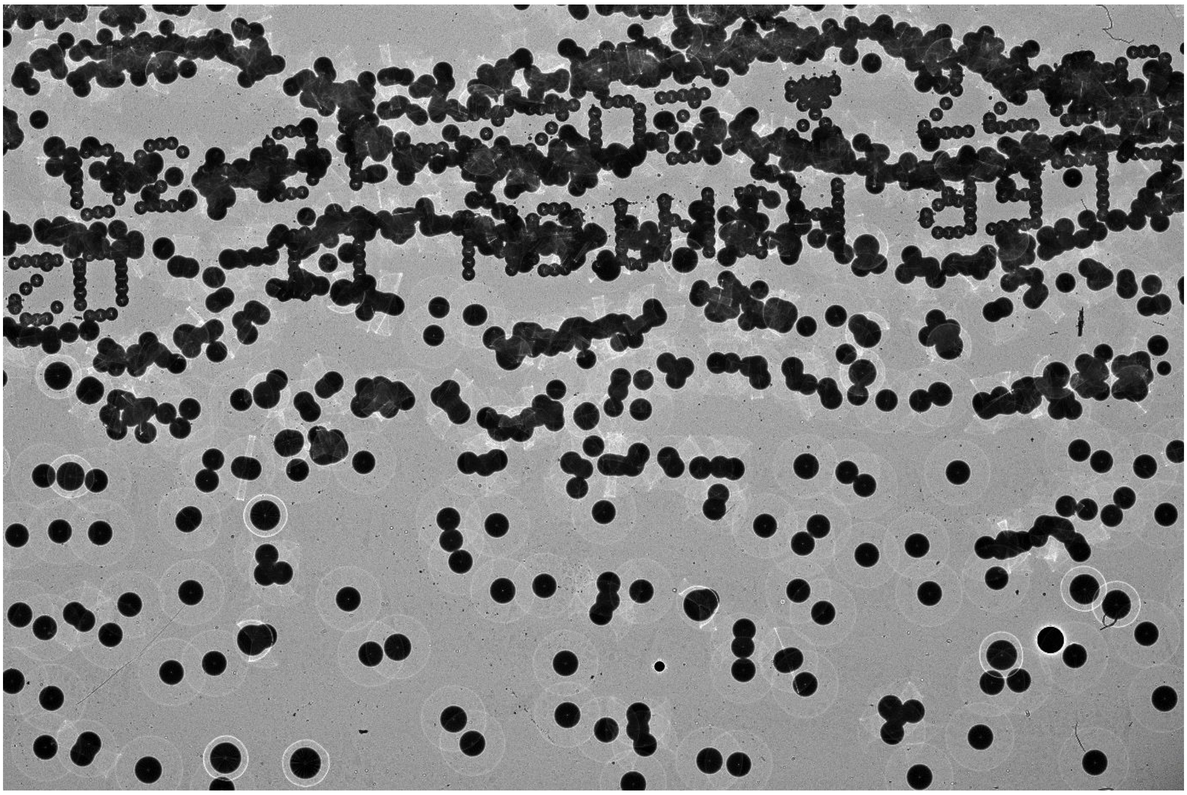

(d)

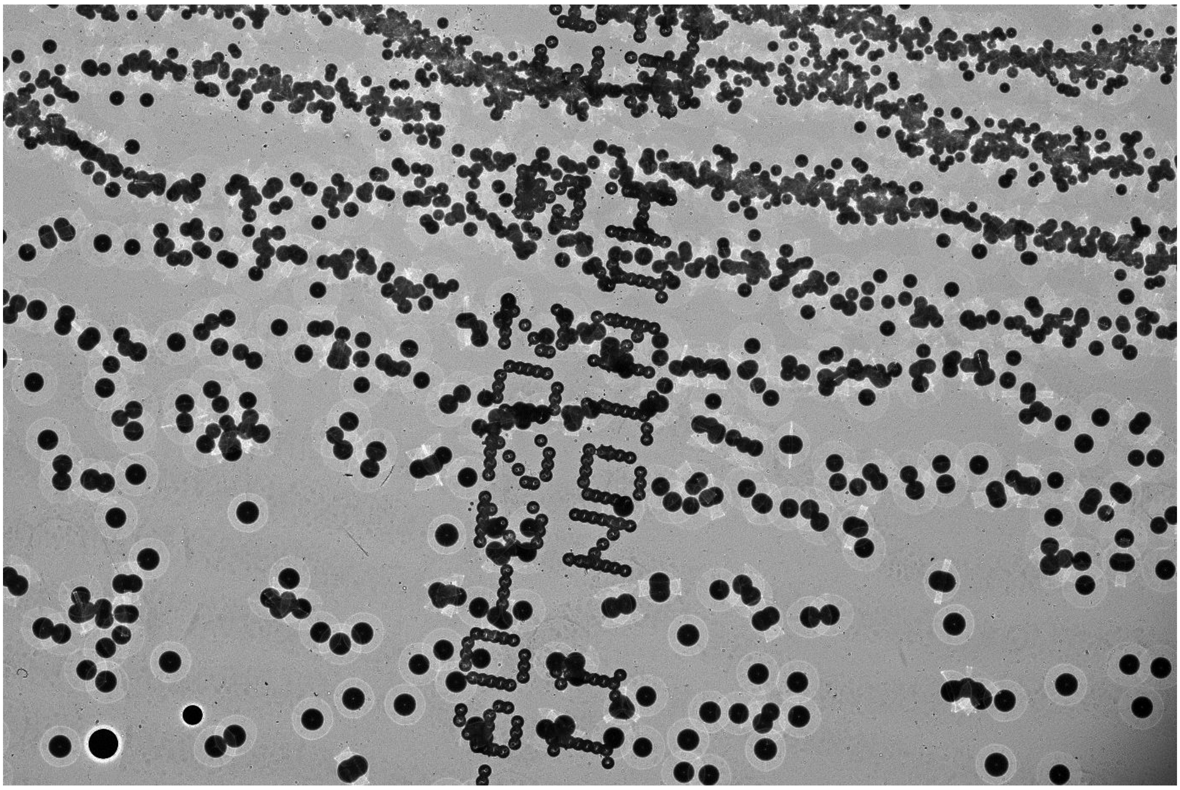

(e)

*Figure S2: Some images of the dataset. (a) E. coli. (b) K. pneumoniae. (c) P. aeruginosa. (d) S. aureus. (e) S. epidermidis. All images are at the same scale, indicated on sub-figure (a). Scale of all images: see Figure 4a.*

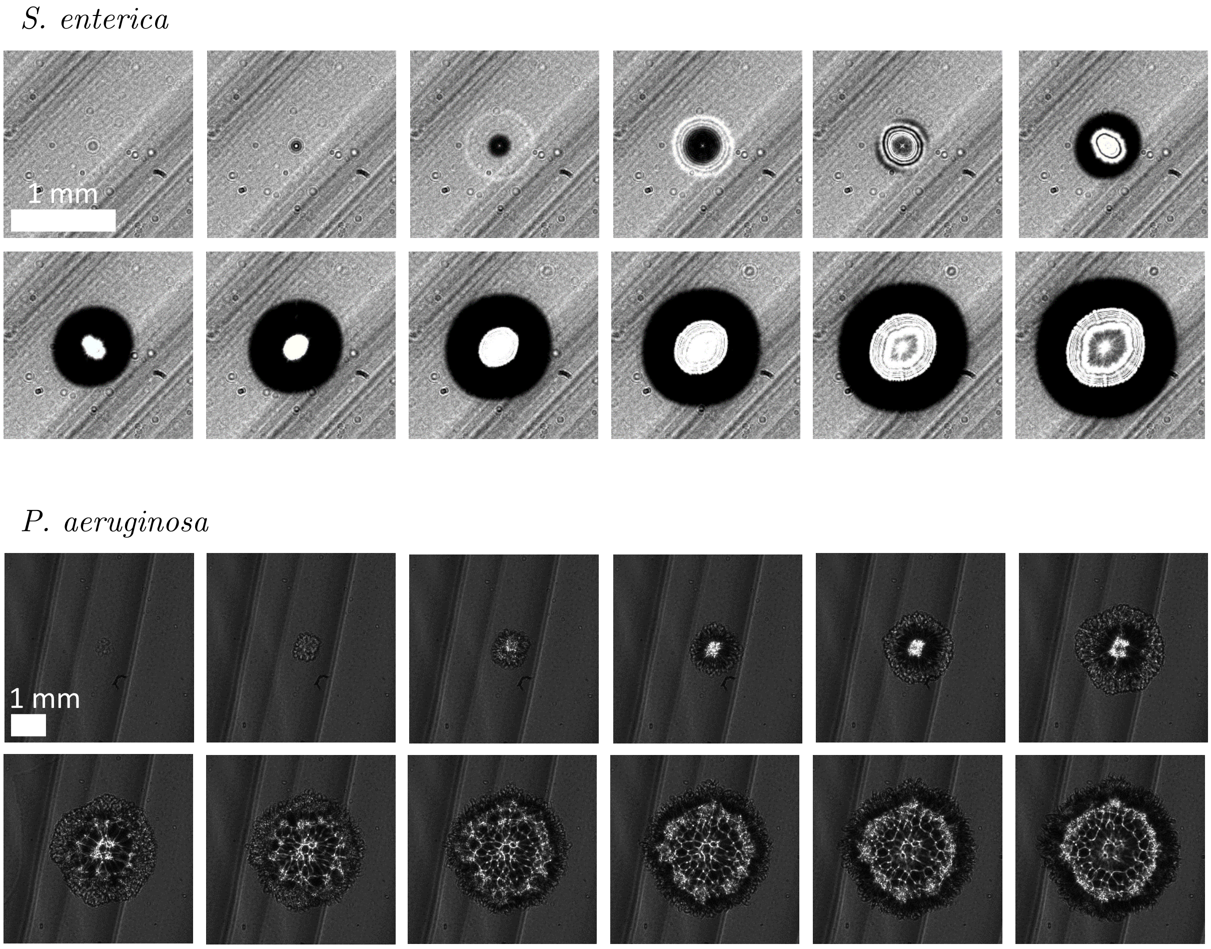


*Figure S3: Time lapses of two colonies showing the evolution of their morphotype over time. The time-lapses start when the colonies first appears, after six hours of growth. The time between two consecutive images is one hour. All images of a given time lapse are at the same scale, indicated on the first image.*

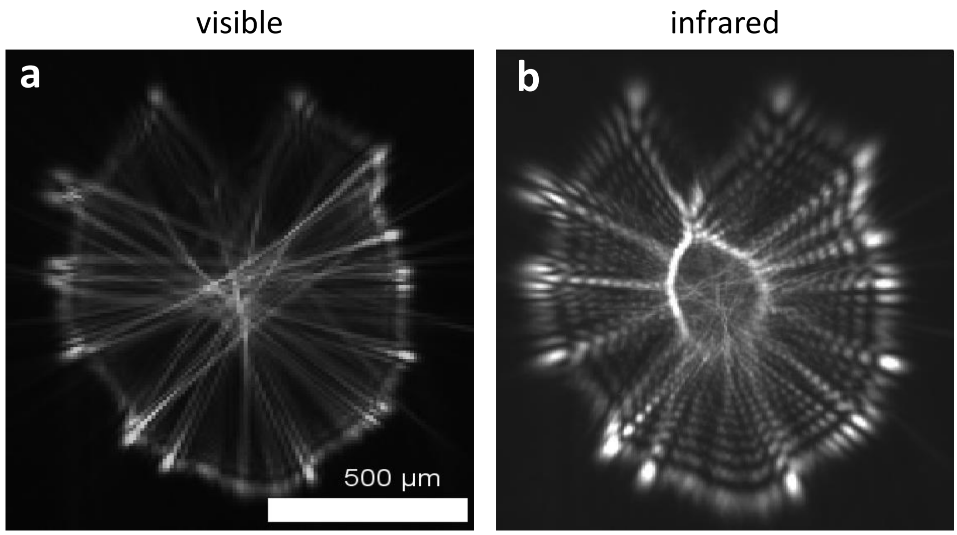


*Figure S4: Wavelength influence. Comparison of the scatterogram of a single E. coli colony when illuminated with (a) visible light (550 nm) and (b) infrared light (940 nm). Both images are at the same scale, indicated on sub-figure (a).*

| species | *E. coli* | *K. pneumoniae* | *P. aeruginosa* | *S. aureus* | *S. epidermidis* | **total** |
|---|---|---|---|---|---|---|
| # patches | 2617 | 2382 | 2239 | 2290 | 1859 | 11387 |

*Table S2: Number of patches per species in the dataset.*

# 3. Machine learning based classification

## 3.1. Hyperparameter optimisation

In the context of a classification algorithm, all parameters describing how the algorithm's training process is carried out are called hyperparameters. We set the value of some of these hyperparameters empirically, but for many others, prior knowledge only narrowed the possible spectrum to a range of possible values. Consequently, as it is frequently done in machine learning pipelines, we carried out an optimal hyperparameter search by assessing the algorithm’s accuracy when run with different hyperparameter values (Table S3). These experiments were conducted on a *validation set*: a small annex dataset composed of samples from preliminary patients.

| Hyperparameter | Tested values |
|---|---|
| Acquisition wavelength | *visible*, infrared, visible + infrared |
| Architecture | ConvNeXt[1], DenseNet[2], EfficientNet[3], InceptionV3[4], *ResNet-18*, ResNet-50, ResNet101, ResNeXt[5], Swin Transformer[6], Wide-Resnet[7], simple CNN |
| Pre-training | *none*, ImageNet, reference strains |
| Data augmentation | none, *flip + rotation*, flip + rotation + distortion + contrast |
| Learning rate | *fixed to* $10^{-3}$, scheduler |

*Table S3: The hyperparameters under study and the values that were tested during the hyperparameter search. Values in italics correspond to the baseline.*

Most of the studied hyperparameters and their values being fairly standard in machine learning literature, we briefly describe the ones more specific to our work:

- (architecture) simple CNN: this architecture, composed of a series of three convolutional blocks followed by two fully connected layers, was destined to assess whether a simple network had enough capacity to perform strongly on the classification task.
- (pre-training) reference strains: Prior to this work, authors from CEA had conducted a related bacterial identification study focusing on reference strains of similar species. We believed that the gathered dataset could be of value to pre-train the network of this work.
- (modality) visible + infrared: corresponding pairs of images acquired in visible and infrared light were concatenated channel-wise. This concatenation was possible because of the almost perfect spatial alignment between visible and infrared images acquired by the system (*i.e.*, no registration was required).

We randomly separated the validation set at the full image scale into two splits (train and test) which were used for all configurations. We set a configuration of hyperparameter values as a baseline (indicated in italics in table S1), and computed the classification accuracy resulting from the change of one hyperparameter value at a time. The underlying hypotheses were that there was no significant interaction between the hyperparameters.

Figure S4 presents the species identification accuracy (mean and standard deviation averaged over ten runs) for each hyperparameter configuration.

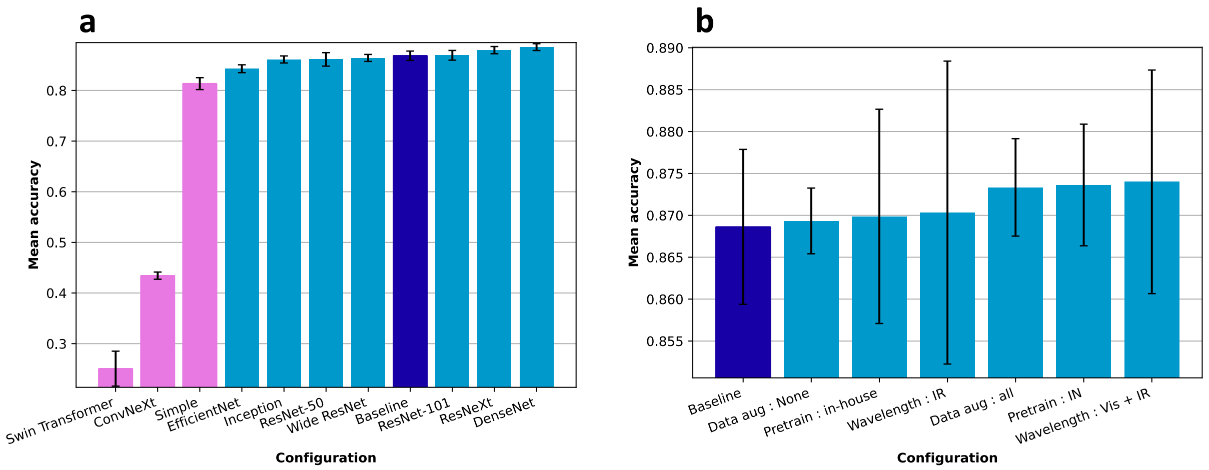


*Figure S5: Mean classification accuracy (y-axis) obtained with the different configurations explored in the hyperparameter search (x-axis). Each configuration corresponds to one hyperparameter variation from the baseline (which is indicated in dark blue). (a) Configurations where the architecture is changed. (b) Configurations where other hyperparameters are changed. Note that the y-axis limits are different between the two graphs. Error bars represent 95% confidence intervals, assuming normal distributions. Results that are statistically significantly different from the baseline are indicated in pink.*

Tukey's test specified that the only significant difference between modalities and the baseline involved configurations with architectures Swin Transformer, ConvNext or the simple network instead of ResNet-18 (pink bars in Figure S3 (a)) (p-value < 0.001). All these configurations led to worse results than the baseline. The performance drop caused by the implementation of modern architectures (ConvNeXt, ViT) was somewhat surprising. We hypothesised that this gap was due either to these architectures being scaled for an amount of data far superior to the one in the dataset at hand, or that their performance more heavily relied on a specific tuning of their distinct hyperparameters. In any case, the small difference between the accuracy reached by the simple CNN and the baseline indicated that the choice of the architecture was not a significant performance bottleneck.

Even more surprising was the fact that no configuration led to a significantly higher accuracy than the baseline, a rather unanticipated result given the breadth of the tested hyperparameters. We hypothesised that classification performance was predominantly limited by *data variability* (strong morphotypic variations of colonies) rather than by *algorithm quality* (suboptimal hyperparameter values). Consequently, we chose the baseline as the optimal hyperparameter configuration as it boasted the desirable side properties of fast training and inference along with low memory consumption. Moreover, its hyperparameter values were all easily implementable in any standard machine learning pipeline, hence enhancing reproducibility.

## 3.2. Patching artefacts

While some of the *E. coli* patches predicted as *K. pneumoniae* showed no obvious patterns that could explain the algorithm's misclassification, a sizable proportion of them seemed to contain too little information to be correctly classified. Indeed, due to the patching process, those patches contained only portions of incomplete colonies with no complete scatterogram (Figure S5). The high proportion of such "incomplete" patches that were misclassified was a further indicator, complementary to the attribution study, of the importance of scatterograms to discriminate between closely related species.

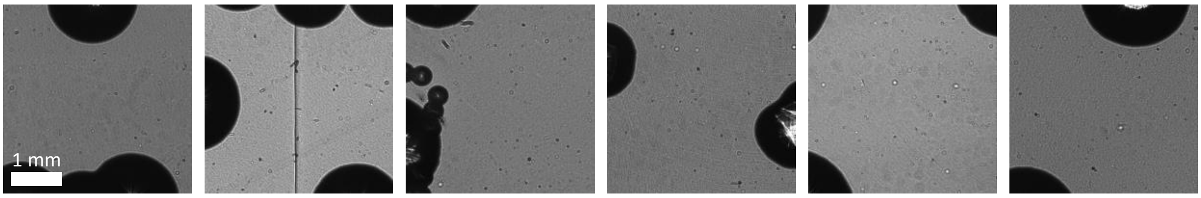


*Figure S6: Some patches of E.coli misclassified as K. pneumoniae showing severe patching artefacts.*

It was not surprising that this scenario only occurred for these two bacterial species. Indeed, colonies from the *Staphylococcus* genus were much smaller and more numerous, and hence much more likely to be completely included in patches. Conversely, colonies of *P. aeruginosa* were typically much larger, leading to patches containing a significant amount of scatterogram information.

These “incomplete” patches underlined the unavoidable complications of any patching process. It would be easy to set the label of such cases as *No colony* by increasing the necessary amount of colony presence for a patch to be labelled as containing colonies. However, doing so would also have resulted in false negatives by the elimination of patches containing smaller *Staphylococcus* colonies, and more importantly does not prevent this case from appearing during inference.

Instead, to mitigate this effect, several more astute patching techniques could be implemented:

- at training and at inference time: implement an automatic patching method where patches, rather than uniformly covering the whole image, are centred on colonies or groups of colonies automatically located by a computer vision algorithm. This algorithm could be focused on the detection of scatterograms (brighter than the rest of the image) or on full colonies (darker than the rest of the image, round shapes). Such automatic localisation would surely face some challenges (possible markings on the Petri dish, variability in the size and density of colonies, in the brightness of scatterograms) but the standardised acquisition conditions (lighting, *etc.*) made possible by the imaging system block out a considerable amount of variability one can encounter in other computer vision tasks.
- at inference time: uniformly patch the whole image, but with an overlap of half the patch size[6]. Carry out inference for each patch as a first step, and then set the final prediction for each overlap as the species with the highest score among all overlapping patches. More elaborate patch blending strategies could be implemented, as they have proved to be effective in biological imaging[8].

# References

1. Liu, Z. *et al.* A convnet for the 2020s. in *Proceedings of the IEEE/CVF conference on computer vision and pattern recognition* 11976–11986 (2022).

2. Huang, G., Liu, Z., Van Der Maaten, L. & Weinberger, K. Q. Densely connected convolutional networks. in *Proceedings of the IEEE conference on computer vision and pattern recognition* 4700–4708 (2017).
3. Tan, M. & Le, Q. Efficientnet: Rethinking model scaling for convolutional neural networks. in *International conference on machine learning* 6105–6114 (PMLR, 2019).
4. Szegedy, C., Vanhoucke, V., Ioffe, S., Shlens, J. & Wojna, Z. Rethinking the inception architecture for computer vision. in *Proceedings of the IEEE conference on computer vision and pattern recognition* 2818–2826 (2016).
5. Xie, S., Girshick, R., Dollár, P., Tu, Z. & He, K. Aggregated residual transformations for deep neural networks. in *Proceedings of the IEEE conference on computer vision and pattern recognition* 1492–1500 (2017).
6. Liu, Z. *et al.* Swin transformer: Hierarchical vision transformer using shifted windows. in *Proceedings of the IEEE/CVF international conference on computer vision* 10012–10022 (2021).
7. Zagoruyko, S. & Komodakis, N. Wide residual networks. *ArXiv Prepr. ArXiv160507146* (2016).
8. Cordier, N., Delingette, H. & Ayache, N. A patch-based approach for the segmentation of pathologies: application to glioma labelling. *IEEE Trans. Med. Imaging* **35**, 1066–1076 (2015).
